# Analytical Approach to Sampling Estimation of Underwater Tunnels Using Mechanical Profiling Sonars

**DOI:** 10.3390/s21051900

**Published:** 2021-03-09

**Authors:** Vitor Augusto Machado Jorge, Pedro Daniel de Cerqueira Gava, Juan Ramon Belchior de França Silva, Thais Machado Mancilha, Waldir Vieira, Geraldo José Adabo, Cairo Lúcio Nascimento

**Affiliations:** Electronics Engineering Division, Instituto Tecnológico de Aeronáutica, Praça Marechal Eduardo Gomes, 50, Vila das Acácias, São José dos Campos 12228-900, SP, Brazil; pdcg@ita.br (P.D.d.C.G.); juan@ita.br (J.R.B.d.F.S.); thaismm@ita.br (T.M.M.); waldir@ita.br (W.V.); adabo@ita.br (G.J.A.)

**Keywords:** profiling sonar, UUV, sampling, mapping, mechanical profiling sonar, Imagenex 881L

## Abstract

Hydroelectric power plants often make use of tunnels to redirect the flow of water to the plant power house. Such tunnels are often flooded and can span considerable distances. Periodical inspections of such tunnels are highly desirable since a tunnel collapse will be catastrophic, disrupting the power plant operation. In many cases, the use of Unmanned Underwater Vehicles (UUVs) equipped with mechanical profiling sonars is a suitable and affordable way to gather data to generate 3D mapping of flooded tunnels. In this paper, we study the resolution of 3D tunnel maps generated by one or more mechanical profiling sonars working in tandem, considering synchronization and occlusion problems. The article derives the analytical equations to estimate the sampling of the underwater tunnels using mechanical profiling sonars (scanning sonars). Experiments in a simulated environment using up to four sensors simultaneously are presented. We also report experimental results obtained by a UUV inside a large power plant tunnel, together with a first map of this environment using a single sonar sensor.

## 1. Introduction

Man-made structures, such as channels and underwater tunnels, are often used in some types of hydroelectric power plants to direct water from the river to the power house. Such tunnels can span from tens of meters to kilometers in length. Engineers must constantly monitor the tunnel conditions in order to prevent tunnel collapse, a catastrophic failure which will interrupt essential services for a long time and lead to major financial losses. While a full collapse of the tunnel is evident, partial collapses deform the ceiling or side walls and often throw debris on the floor. The detection of such features is key to the inspection of these tunnels.

Inspections of such power plant tunnels can be very challenging. Typically Unmanned Underwater Vehicles (UUVs) only access these tunnels when the power plant operation is halted for scheduled maintenance, due to the risks of operating the vehicle with a high water flow [1]. This constraint means that there is limited time for testing the UUV operation in the power plant tunnel before its deployment. Furthermore, water visibility in such tunnels is often very limited. During our experimental tests, water visibility was between 1–3 m (in clear rivers water visibility is around 20 m [2]) and in these conditions the use of sonars is a viable and affordable alternative. Some authors have reported success using active optical three dimensional (3D) scanners and other types of high-end commercial equipment [3,4] to reach 10 to 20 m of range under clear water conditions. In our case, water turbidity would restrict laser or vision based range finders to close range operation [3] and therefore they could not be used for tunnel mapping.

There are mainly two broad groups of sonars—multi-beam sonars (MBSs) and mechanical profiling sonars (MPSs). MBSs can cast several rays at the same time, collecting range data at several orientations at once, while MPSs usually cast a single ray from a sensor spinning under the control of a mechanical device. MPSs and MBSs typically have a configurable angular step between adjacent samples, which often change the pooling speed of the sensor. Both types of sensors can provide range data to map underwater environments. However, the range sampling frequency for MBSs is often orders of magnitude higher than MPSs. On the other hand, the price of an MBS is often several times the price of a MPS. Therefore using multiple MPSs [5] might be an affordable alternative solution.

In this paper we study the mapping of flooded tunnels using an MPS, or a combination of them. In particular, we focus on the effect of UUV velocity, sensor angular velocity and step size on the distance between sampled points obtained with range readings from a pool of several MPSs, testing two different spatial configurations of sensors—sector offset and phase-shifted continuous rotation. We use a commercial product, the Imagenex 881L profiling sonar, as a reference for sensor-specific settings of angular velocity and steps. Experiments were performed in simulated versions of two robots equipped with 881L sensors. We show how to predict the distance between samples on the surface of the tunnel for one or more sensors in order to properly select the sensor configuration, which often has several parameters that cannot all be tested on site. We also present our first test inside an actual tunnel considering one single sensor to validate its use in large tunnels.

## 2. Related Work

In the context of tunnel mapping, Hosko [1] reports lessons learned over a five year case study of inspection of hydro powers, emphasizing the problems to inspect tunnels with divers, the cost of dewatering long tunnels, and pointing the need for mapping such structures using UUVs.

In the context of vision based sensors, plenty of work is currently being undertaken to achieve vision in turbid waters. Range gated underwater laser imaging [6,7], integral image techniques [8,9], synthetic confocal imaging [10] and stereo cameras together with structured light [11] have shown to work in turbid environments, even though only for close range applications [3]. Bleier and Nüchter [4] even highlight a commercial sensor which can work in the range of up to ten meters under specific water conditions. Still, the visibility inside the tunnel was usually between 0.5–3 m. While such visual based methods can certainly be used to map certain types of tunnels, the dimensions of the tunnels we are working exceed the ranges reported by such methods and the range reported by commercial devices is only possible with water conditions which are not present in our environment. In fact, even though many visual based methods can offer better refresh rate and even resolution at close range, sonars are still the norm for a number of underwater applications [3]. For smaller tunnels, visually based methods are the best option.

Drap et al. [12] fuse vision and sonar data to generate accurate representations of underwater environments with an ROV; however, in our case we cannot use vision, since water turbidity is high. Fairfield et al. [13] reconstructs a cenote using sonars with one meter voxels, comparing SLAM approaches and dead reckoning, and pointing the limitations of sonars beams, which often cannot provide enough resolution for feature detection of large environments such as cenotes. The voxel size is set to one meter, but the discussion focuses on the beam shape, while information on how the voxel size was defined is not further detailed. While the resolution of the map may not be important for environmental surveys of cenotes, the resolution of the map is critical for the inspection of flooded tunnels. Fairfield et al. [14] presents a 3D SLAM method for mapping structures based on a 2D Rao-Blackwellized Particle Filter [15]. The study focuses on memory and computational costs when increasing the voxel size, which show cubic growth with. Lasbouygues et al. [16] presents a robotic system which is able to perform the mapping of karst aquifers with an ROV and also an underwater scooter operated by divers. They show that these two proposed systems can perform the task. White et al. [17] present several mapping strategies using a UUV inside the flooded regions of cisterns at Malta, including sonar mosaicking and SLAM with the aid of a tether counter—when walls are not able to provide sufficient features to localize the robot along the cistern length. McVicker et al. [18] also presents the mapping of cisterns using an ROV and stationary scans in combination of a SLAM approach to achieve 3D reconstruction. Another work involving cisterns is presented by Dobke et al. [5], which uses two orthogonal mechanical sonars and the Iterative Closest Point (ICP) algorithm to map cisterns, showing we can combine MPSs in tandem in underwater environments. Other relevant works on mapping cisterns include the works of Clark et al. [19,20].

Mallios et al. [21] presented a pose SLAM algorithm based on techniques derived from the ICP algorithm to localize the SPARUS AUV inside a natural underwater tunnel. Stipanov et al. [22] presented the mapping of a cave using a tethered ROV with a resolution of two meters. Their experiments show that a tether can impair the forward speed and even stop the UUV due to friction against rocks. While DVLs are an alternative for estimating the UUV velocity inside tunnels, Kantor et al. [23] point out that the dropout rate of the DVL can affect localization, even in outdoor environments which can use GPS. In a seminal study, the Tunnel Sea Lion ROV design [24] is presented, including functions such as the inspection of tunnels. Zhou et al. [25] present the design of a hybrid AUV/UUV for the inspection of power plant tunnels along with tether counter and heading control data. Loisy et al. [26] report a first inspection of long underwater tunnels with 2.4 m of diameter using an ROV. The diameters of such tunnel is smaller than those we are working on, 10–20 m. They report counter current, which we also faced, due to a leak, considerably affecting maneuverability and the operation. In the present work, we report our experience while mapping an underwater tunnel with large dimensions, including information about the visibility on site and the environment we have faced.

In addition, the quality of sampling of systems using one or more synchronized MPSs in tandem in simulated scenarios is studied. Information theory is the foundation of the present work. We use it to formally estimate the spatial distance between samples—specially the sampling period and the sampling theorem [27,28,29]. One can see the process of robotic mapping as a signal reconstruction process, where the environment surface can be seen as a signal of interest, and the time *t* is replaced by the space covered by the robot and its sensor over the region which must be reconstructed. Likewise, any range sensor can be seen as a wave used to sample part of such signal as the robot moves over the terrain.

For the simulations, we have considered different simulation frameworks [30,31,32,33,34,35,36,37,38,39]. More recently, Paravisi et al. [40] presented a new simulator, usv_sim_lsa, based on UWSim [32], improving support to USVs and realistic environmental disturbances. Many of the simulators studied by the authors also work for UUVs. They point out problems in several simulators, including lack of documentation and running code. Manhães et al. [41] present the uuv_simulator, which considers factors such as added mass in the robot motion. Among the available simulators, we have selected two: usv_sim_lsa and the uuv_simulator, both which work with ROS and Gazebo. While the former offers a richer set of environmental simulations and better underwater rendering, the latter offers slightly better representation of the robot motion in water at the cost of increased instability. We stood with the uuv_simulator, mainly due to its more complete motion model.

The two prototypes we have designed, VITA 1 and VITA 2, were ported to the uuv_simulator, including sensors, to test synchronized MPSs. We also recreate an approximation of the underwater tunnel scenario we are currently working with.

## 3. Formalization of the Mapping Problem

In this section we mathematically describe an ideal robot designed to perform mapping of a partially structured man-made tunnel and its ideal mapping sensor used to formalize the problem of mapping for one sensor connected to a robot, as the latter moves with constant velocity, v.

**Figure 1:**
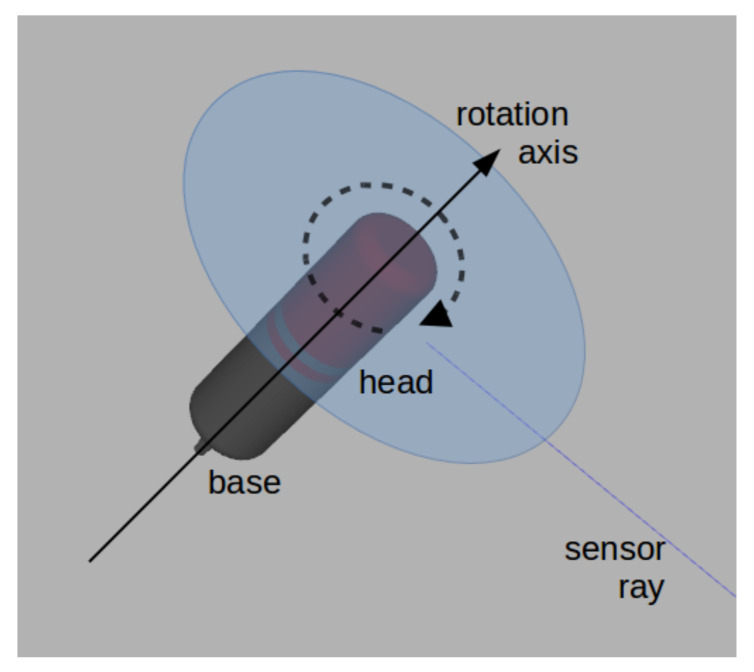
An example of profiling sensor with a single ray connected to a moving head.

We start with a sensor constructed with a fixed base connected via a revolute joint to a spinning head, where it is fixed (see Figure 1). The head rotates in discrete and uniform angular steps around the rotation axis, represented by the normalized vector $\hat{r}$, with constant but configurable angular velocity, ω. The exterioceptive sensor is a single point sensor which samples points at each angular step, θ, with heading $\hat{s}$, orthogonal to $\hat{r}$ such that $\hat{s}\cdot \hat{r}=0$.

**Figure 2:**
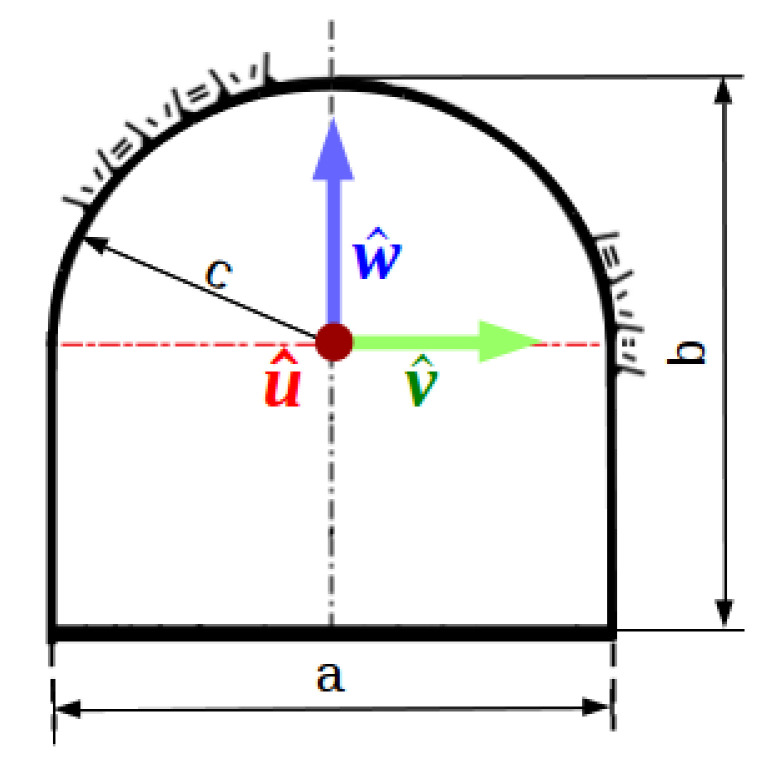
An example of tunnel cross-section with base with length *a*, the tunnel height is *b* and its arc shaped ceiling has radius *c*. Vectors $\hat{u}$, $\hat{v}$ and $\hat{w}$ are the normalized basis vectors of the tunnel reference frame, where $\hat{u}$ is orthogonal to the cross-section of the entrance of the tunnel.

The sensor is mounted in a UUV with normalized heading vector, $\hat{h}$, matching **r**, such that $\hat{h}\cdot \hat{r}=1$. In addition, we position the UUV at a point, P=(0,0,0), centered at the entrance of a tunnel with cross-section shown in Figure 2, where *a* is the tunnel width, *b* is the tunnel height, and $\hat{u}=\left(1,0,0\right)$, $\hat{v}=\left(0,1,0\right)$ and $\hat{w}=\left(0,0,1\right)$ form the basis of the coordinate frame of the tunnel. We assume the tunnel is straight and, as the robot moves, it is always at a point $P_{x}=\left(x,0,0\right)$ inside the tunnel, where 0≤x≤l and *l* is the tunnel length along $\hat{u}$. The robot heading, $\hat{h}$ is co-directed with $\hat{u}$, making every sensor ray co-planar with a plane represented by point $P_{x}=\left(x,0,0\right)$ and its normal $\hat{u}$, where a cross-section along the tunnel length lies.

We then start to study the resolution of 3D maps of tunnels. We break the problem into parts. First we consider the cross-section and then the whole map as the robot moves along the length of the tunnel with constant velocity. Lastly, we study the behavior of multiple sensors working in tandem.

### The Sampling Period over Cross-Sections

When our ideal point-wise robot is stopped, we can disregard the effect of the robot motion over $\hat{u}$ and simply analyze the behavior of the sensor as it rotates. Therefore, we can devise a function *g* representing the cross-section of the tunnel in cylindrical coordinates as

(1) g(t)=〈x,ρ(t),α(t)〉,

where at any time *t*, *x* is the value that designates the point $P_{x}=\left(x,0,0\right)$ where the robot is stopped, ρ(t) is the distance from the center of the cross-section to the internal surface of the tunnel at angle α(t), all on the plane with normal $\hat{u}$, regardless of *x*. This way, ρ(t) conveniently represents the sensor range reading, while α(t) represents the current angle of the head of the sensor—assuming the robot and sensor are centered at the cross-section (see Figure 2).

If using Cartesian coordinates, the function g(t) can be written as

(2) g(t)=〈x,ρ(t)cos(α(t)),ρ(t)sin(α(t))〉.

Even if there are no occlusions blocking the sensor field of view, sensors do not have infinitely small step angles. The number of angular steps for a full rotation of the sensor, *n*, is finite and given by

(3) $$n=\frac{2\pi }{\theta },$$

where θ, the sensor step angle, is often small (typically around one degree), depending on sensor configuration and quality.

Since the profiling sensor offers a single range measurement of the tunnel cross-section at each angular position, α(t) and ρ(t) are only available at time instants $t=kT_{s}$, where

(4) $$\alpha \left(t\right)=\alpha_{0}+\omega t,$$

and k=0,1,…,n−1, $T_{s}$ is the sampling time (time interval between successive samples), and $\alpha_{0}$ and ω are respectively the starting angle and the angular velocity of the sensor head. Consequently, only a sampled version of the function g(t) can be acquired in practice.

Angular sampling in our case produces evenly spaced angles, but spatial samples are usually not evenly spaced. The distance between samples in space will only be equal if the robot is mapping an ideal duct, pipe or tunnel with the shape of a cylinder. In this case, the cross-section of the environment is a circle and the space between samples is guaranteed to be the same. For our tunnel, if c=b/2, the ceiling respects such condition; still, at the floor and vertical walls, it is not. Moreover, the reconstruction, simply put as a Fourier transform used to reconstruct a signal, would also rely on base functions of different frequencies, but such frequencies represent changes in the terrain or cross-section, measured by the rate of change of ρ, in meters. The information between such samples can be lost if the features at the surface are not salient enough or are smaller than twice the distance between samples—which is a consequence of the sampling theorem. Therefore, we can lose data with our ideal sensor.

**Figure 3:**
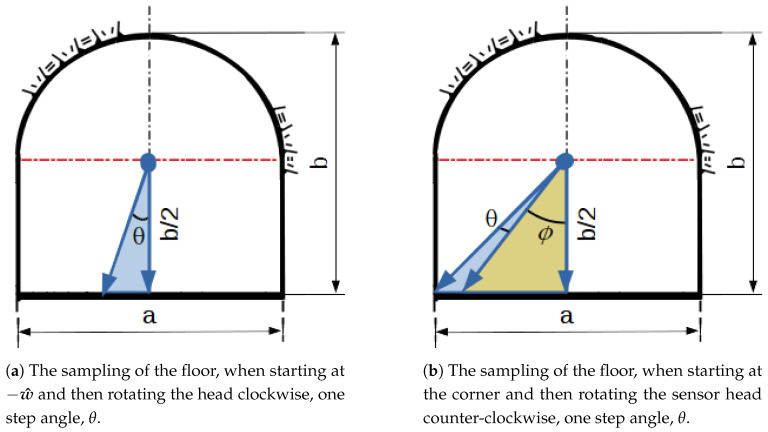
Analysis of floor samples in the two extreme cases: (**a**) when the sensor is centered and perpendicular to the floor; and, (**b**) when the sensor hits the corner.

In order to analyze the sensor while it is mapping the floor, we keep the initial angular position with the sensor pointing down to $-\hat{w}$ (see Figure 3). The distance between samples, $T_{y}$, when the sensor rotates clockwise one step angle, θ, is trivial and given by

(5) $$T_{y1}=∥\frac{b\tan(\theta )}{2}∥.$$

This is the spatial sampling interval along the cross-section with the smallest distance between the sensor and the floor. Besides, the distance between samples on the floor is proportional to the tangent of the angular position of the head, α, and the angular step, θ. On the other extreme, assuming the sensor moved θm steps clockwise, conveniently reaching the corner which connects the floor and side wall (see Figure 3), the sampling interval is given by

(6) $$T_{y2}=∥\frac{b(\tan(m\theta )-\tan\phi )}{2}∥,$$

where ϕ=(m−1)θ. It represents the distance between samples with the largest distance between the sensor and the floor—b(tan(mθ)=a/2 when *m* reaches the corner. Nevertheless, Equation (6) also describes all the distances between consecutive samples on the floor by changing the value of *m*. In fact, it reduces to 5 when m=1. Now we can write Theorem 1 (see proof in Appendix A).

**Theorem** **1.** *When a single point sensor rotates around an axis to perform the sampling of a flat region, sampling with uniform angular steps results in non-uniform spatial sampling. In particular, if we assume the angular sampling starts at $\theta =0^{{}^{\circ}}$ forming a vector which is perpendicular to the flat region, any pair of samples on the floor, obtained by stepping from m(θ−1) to m(θ), will display a smaller spatial distance between samples than the next pair obtained at the next step—that is, going from m(θ) to m(θ+1).*

Thus, we can infer the best and worst cases of spatial sampling over the cross-section at flat regions with direct impact on sampling, knowing exactly where poor sampling will occur. That is, $T_{y1}$ corresponds to the smallest distance between samples on the flat region, while $T_{y2}$, when the sensor ray hits the corner, is the largest. In other words, the best resolution obtained while sampling the floor would occur at the center of the tunnel, degrading as sampling approaches the corners

Once the robot moves sideways, the distance to the farthest corner is increased in the same amount. While such displacement to the sides can be small, its resulting effect in the tangent function may not be negligible, depending on the size of the tunnel under consideration and θ. Similarly, once the robot moves vertically towards the floor, the distance between adjacent samples will decrease when the ray is nearly perpendicular to the floor. However, it will increase near the corners, increasing drastically as the ray approaches parallelism with the floor. Finally, if the robot does not keep its heading aligned with the length of the tunnel, the difference in the yaw angle from the correct heading will also result in an increased distance from the farthest wall. As a consequence, the values of $T_{y1}$ and $T_{y2}$ are affected by the robot displacement and attitude.

Sidewalls can be compared applying the same strategy, if they are flat. Then, the last step is to study the ceiling. For tunnels with a rectangular cross-section, there is nothing else to do but comparing such regions. However, for the cross-section with the circular shaped ceiling of Figure 2, the spatial distance between samples is given by

(7) $$T_{c}=∥b\sin(\theta /2)∥,$$

in the specific case where the center of the cross-section, matches both the robot position and the center of the half-circle which forms the ceiling, that is, c=b/2=a/2. The distance between samples in this case is always smaller at the ceiling than the floor. That is, as long as the profile of the ceiling presents a convex shape and the ceiling curvature does not go beyond the center of the cross-section, $T_{\rfloor }<T_{y1}$. The intuition behind it is this: if the ceiling is convex, every time the sensor spins one step angle, the ceiling curvature keeps the measurement ρ constant or smaller than the distance of a purely flat ceiling parallel to the floor (see Appendix B). Besides, the equation for the ceiling is bounded by the sine function, which can only grow up to 1, while the tangent function which defines $T_{y2}$ can grow to infinity.

Therefore, the sampling quality along the plane belonging to the cross-section is constrained by corners. Such sampling period is given by $T_{y2}$. $T_{c}$ and $T_{y1}$, can both be used to as best case scenarios of sampling along $\hat{v}$. However, $T_{c}$ calculation varies with ceiling shape and distance, since the ceiling often has specific constructions which do not match our design. Thus, using the simple floor equation may be a reasonable approximation.

Note, that such distances also depend on the robot position, and small changes to the sides lead to changes in minima and maxima. Also, man-made constructions often have sharp corners where the derivative of the function representing the surface is not defined. Such sharp corners demand infinite samples, previous knowledge about the terrain or some sort of heuristic for reasonable reconstruction. Such discontinuities usually are detected by sudden increases in the distance between adjacent samples, which change maxima values. Discontinuities in the surface will also reflect on minima, since a salient object, for example, a rock, may lead to the reduction of the distance between the sensor and the feature.

We can also compute the mean distance of consecutive samples on the floor. One way to compute it is using Equation (6) in the form of a sum, which for the first quadrant is:

(8) $${\bar{T}}_{y2}=\frac{\sum_{k=1}^{m}∥\frac{b(\tan(k\theta )-\tan((k-1)\theta ))}{2}∥}{m}.$$

Note that Equation (8) is a telescopic sum which can be directly reduced to:

(9) $${\bar{T}}_{y2}=∥\frac{b(\tan(m\theta )}{2m}∥=\frac{a}{2m}.$$

Since we have a symmetry, both sides of the flat region reduces to the same equation.

Moreover, we can estimate the mean distance between samples over the entire cross-section as

(10) $${\bar{T}}_{y_{p}}\leq \frac{p}{n},$$

where *p* is the perimeter of the cross-section and *n* is the number of angular steps given by the sonar in one revolution of the sensor (see proof in Appendix C). Note that only ${\bar{T}}_{y_{p}}$ is independent of the position of the sensor over the cross-section.

Together, ${\bar{T}}_{y_{p}}$, $T_{y2}$$T_{y1}$ (or $T_{c}$) can be used to estimate the size of features which can be detected over the cross-section. Still, the robot forward motion affects sampling in important ways.

## 4. Considering Forward Motion

**Figure 4:**
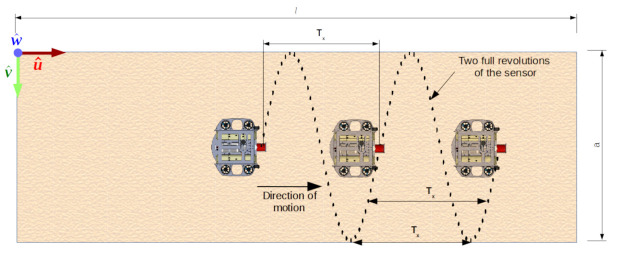
The impact of velocity on mapping with single point sensor when a robot moves with constant linear velocity v=(v,0,0).

In order to map the tunnel, the robot must move over the environment—that is, over axis $\hat{u}$. In our analysis we consider the robot velocity v=(v,0,0), that is ∥v∥=v. Thus, no single point belongs to the same cross-section, as we have previously considered (see Figure 4). For instance, if the robot moves over a tunnel in the shape of a cylinder with constant speed, the resulting point cloud will form points over a spiral.

That is, the distance between consecutive point samples on the tunnel over axis $\hat{u}$ (along the tunnel length) will be constant and given by

(11) $$d=vT_{s},$$

where $T_{s}$ is the time taken for the sensor to move one angular step, θ. Since sampling is uniform over such axis, the sensor always gives *n* steps per revolution of period, *T*—that is, the time it takes for the head to get back to the same position is *T*, while the distance between consecutive samples over $\hat{u}$, taken at the same angular position, is given by

(12) $$T_{x}=Tv,$$

which is, in fact, the spatial sampling period over $\hat{u}$. Note that, unlike the derived sampling periods for the cross-section, $T_{x}$ does not depend on the distance, ρ, measured by the sensor. Thus, when v→0, $T_{x}\to 0$. The smaller $T_{x}$, the better the resolution is over $\hat{u}$. In fact, $T_{x}$ defines the maximum size of anomalies which can be detected along the tunnel length. For instance, a rock with *s* meters of length might not be detected on the floor if $T_{x}\geq s$, due to sampling problems. In order to minimally detect the presence of the rock at the floor, we have to make $T_{x}\leq s/2$, which is a direct consequence of the Sampling Theorem. The detection of such anomalies is critical for inspection, since missing one means we are potentially losing information about an imminent collapse of the tunnel.

Of course, $T_{y2}$ is also affected by the small displacement *d*, resulting from Equation (11), due to robot motion. Therefore, the distance between consecutive samples is given by:

(13) $$T_{y_{max}}=\sqrt{d^{2}+T_{y2}^{2}},$$

at the corners. Similarly, as the distance between consecutive samples changes, the minimum distance on the floor and on the ceiling are also affected in the same way by *d*, resulting in:

(14) $$T_{y_{min}}=\sqrt{d^{2}+T_{1}^{2}},\;\;\;\;T_{y_{min}}=\sqrt{d^{2}+T_{c}^{2}}.$$

Note that, the minima will often occur at the ceiling, but the selection of equation to be used as reference can be dependent on the desired end use.

The mean is also affected, but the displacement over axis is $\hat{u}$ is md, since the head is moved *m* times to go from the center (perpendicular ray in relation to the floor) to the corner, which results in:

(15) $${\bar{T}}_{y}=\sqrt{{\left(nd\right)}^{2}+{\left(T_{y_{p}}\right)}^{2}}.$$

**Figure 5:**
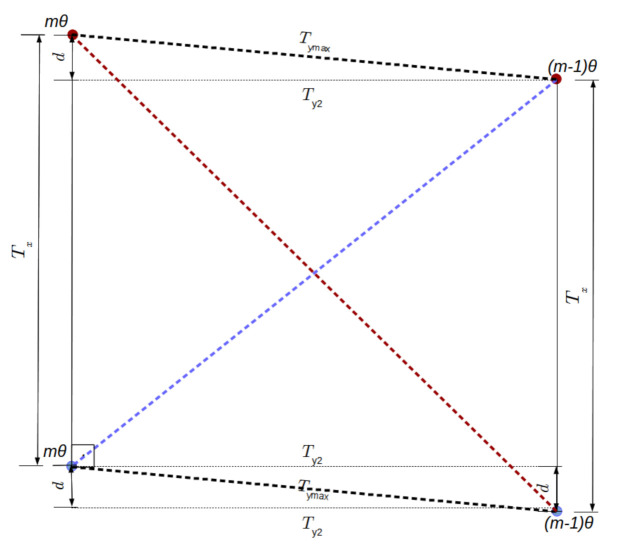
Distance between pair of sensor readings which are closest to the corner, mθ and (m−1)θ angular positions, during the first sensor revolution are displayed as blue vertices, while the same samples at the next revolution are in red. Note that they form a parallelogram of sides $T_{x}$ and $T_{y_{max}}$, with the largest distance between samples at the parallelogram at the largest diagonal, shown in red dashed lines.

We know $T_{y_{max}}$ and $T_{x}$ do not form a straight triangle due to the robot displacement between sensor readings. In fact, the pair of samples obtained from mθ, at the corner and (m−1)θ, one sample before reaching the corner, and the next pair of samples at the same head position but subsequent period of revolution together make a parallelogram (see Figure 5).

Such a parallelogram defines the worst case of distance between samples, which is not perpendicular to any axis. Instead it is at the largest diagonal of the parallelogram. The size of such diagonal is given by:

(16) $$T_{max}=\sqrt{{\left(T_{x}+d\right)}^{2}+{\left(T_{y2}\right)}^{2}}.$$

Features may not be detected by the sensor of if they are smaller than $2T_{max}$.

**Corollary** **1.** *Given a tunnel with convex and symmetric ceiling, and a robot centered at the cross-section, moving with constant speed along the tunnel length and equipped with a spinning profiling sensor, the largest distance between samples obtained by such system is T, given by Equation (16).*

Likewise, the mean can be computed as

(17) $$\bar{T}=\sqrt{{\left(T_{x}+d\right)}^{2}+{\left({\bar{T}}_{y}\right)}^{2}},$$

while the minimum distance is given by

(18) $$T_{min}=\sqrt{{\left(T_{x}+d\right)}^{2}+{\left(T_{y_{min}}\right)}^{2}}.$$

Note that when the robot moves slowly, as is the case with many UUVs, the covered distance in the time between consecutive samples is often negligible, given the angular velocity of the sensor. Therefore, *d* can be set to zero and ignored. Also, Equation (16) which represents the parallelogram is simplified to the rectangle formed by $T_{y1}$ and $T_{x}$. Thus, $T_{y_{min}}\approx T_{c}$ (or $T_{y1}$), $T_{y_{max}}\approx T_{y1}$ and ${\bar{T}}_{y}\approx T_{y_{p}}$. This is also true for Equations (17) and (18). Interestingly, this is the case for an MBS and laser based profilers as well, which means that they will suffer sampling problems similarly along the cross-section, but for those sensors, usually $T_{x}$ are considerably smaller than that of MPSs.

## 5. Multiple Sensors Strategy

A single point sensor such as that idealized in the previous section may suffer due to poor sampling along the tunnel length. Multi-beam profile sonars with a field of view of $360^{{}^{\circ}}$ can sample many points at the same time, decreasing the size of $T_{x}$, which avoids the spiral pattern generated by single point sensors. However, such sensors are expensive. A less costly approach is to combine single point sensors, which can potentially decrease the distance between samples by a factor equal to the number of MPS.

However, some decisions have to be made regarding the way each sensor will work. Recall that the cross-section is separated into *n* uniform angular steps—that is, nθ=2π. Now, suppose we have *s* sensors, $s_{0},s_{1},\cdots ,s_{s-2},s_{s-1}$. We can divide the field of view of the sensor into equally discrete parts, 2π/s∈R and each sensor can be responsible for its own interval

$$\frac{2\pi k}{s}\leq j<\frac{2\pi (k+1)}{s},$$

where k={k∈Z|0≤k≤s−1} for each sensor $s_{k}$. In this setup, all sensors move from $\frac{2\pi k}{s}$ to $\frac{2\pi (k+1)}{s}$ and then back continuously. Another approach is to keep all sensors spinning continuously with a difference of phase to φ(k)=2πk/s for each sensor. While these two approaches look similar, they are not. We discuss the two approaches below, starting with phase shift.

### 5.1. Phase Shift

If we keep all sensors spinning but with different phases, each sensor will reach a given angular position with a difference of phase given by φ(k)=2πk/s, where k={k∈Z|0≤k≤s−1} is the id of the sensor, for each sensor $s_{k}$. Therefore, every T/s seconds a sensor will reach the same head position, leading to a constant uniform sampling of $T_{x}/s$ over the length of the tunnel.

**Figure 6:**
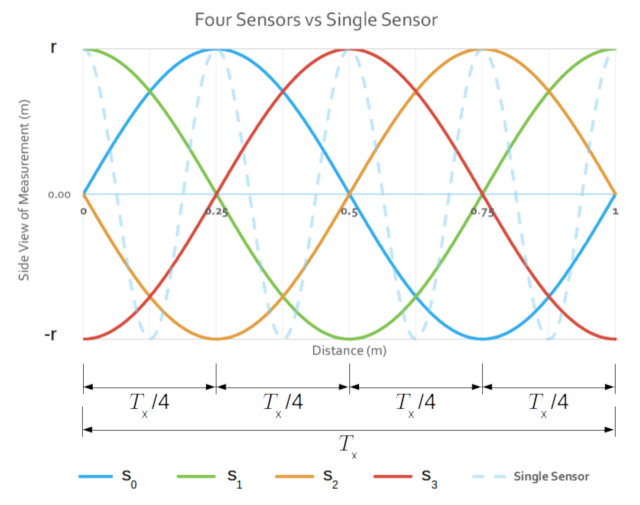
The behavior of four identical sensors $s_{0},\;s_{1},\;s_{2}$, and $s_{3}$, with phases φ(k) for each sensor $s_{k}$—that is, the phase for each sensor is $0^{{}^{\circ}}$, $90^{{}^{\circ}}$, $180^{{}^{\circ}}$ and $270^{{}^{\circ}}$ respectively. Samples at the same head position for all sensors are out of phase and the distance between subsequent samples with the same head position is always $T_{x}$/s, where *s* is the number of sensors. For all sensors, the distance covered while performing one full sensor revolution is $T_{x}$. A 4x faster sensor is shown in blue dashed lines. As the slow sensors can sample 4 points at the same time, sampling does not happen exactly at the same points of a faster sensor, but the distance between samples are the same—for example, the floor is sampled with the same spatial distance, but not at the same points.

Let’s suppose we are working with four identical sensors, that is s=4 with sensor $s_{0},\;s_{1},\;s_{2}$, and $s_{3}$, attached to our ideal UUV, where, as before, all the sensors are at the center of the cross-section of an ideal cylinder-shaped tunnel with radius *r* and length *l*. The robot is traveling with forward speed, *v*, so all the sensors have $T_{x}=1$ m with phase φ(k). Therefore, the phase for each sensor will be $0^{{}^{\circ}}$, $90^{{}^{\circ}}$, $180^{{}^{\circ}}$ and $270^{{}^{\circ}}$. Figure 6 shows the side view of the sampling of the surface of such ideal tunnel, where the distance between samples at the floor and at the ceiling for all sensors is shown to be $T_{x}/s$. The same behavior happens for all samples at the same head position for each of the sensors.

**Figure 7:**
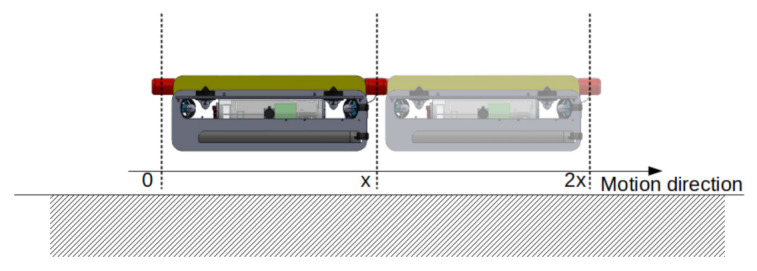
Impact of sampling with two sensors when the robot moves with constant velocity v=(v,0,0) and the distance between sensors is *x*. When $s_{1}$, reaches the position of $s_{0}$, phases must be shifted by $180^{{}^{\circ}}$.

If we consider all sensors at the same position in space, we can ignore positioning of sensors and apply the specified phase shift for each sensor. However, two MPSs cannot occupy the same position in space. Thus, the phase difference must be φ(k)=2πk/s, where *s* and *k* are given as before, but it must happen when the sensors which are behind reach the same position (see Figure 7) of the forward reference sensor $s_{0}$. That is, if the forward speed of the robot is *v* along the tunnel length, we need the phase difference to be φ(k) after *x* meters, which takes t=x/v seconds. Then, if the reference sensor $s_{0}$ starts with angular position $\alpha_{0}=0$ at position x, and $s_{k}$ is also with $\alpha_{k}=0$, but at position 0, when $s_{k}$ moves from 0 to *x* its head position will be given by

(19) $$\alpha_{k}=\omega x/v,$$

where ω is the angular velocity for all the sensors. Therefore, for the sensors which are behind to be in the desired phase at *x*, their head positions must start with a phase shift of

(20) γ=φ(k)−ωx/v,

in order to cancel the phase shift caused by speed and apply the phase between the position of the head of each sensor.

However, if the sensor $s_{k}$ is pointing at the opposite direction of the reference sensor $s_{0}$, its head position α will increase rotating to opposite directions. Thus, the phase shift for sensor $s_{k}$ is given by

(21) γ=2π−(φ(k)−ωx/v).

Thus, we may control the size of $T_{x}$ by changing the forward speed *v* to achieve the desired result. Nevertheless, increasing *v* will increase the distance between samples along the length of the tunnel, which may not be desirable, while decreasing it may take too long to complete the mission.

Note also that we can design a robot so that the distance between the forward sensor, $s_{0}$, and the distant sensor, $s_{k}$, is x=vT or x=qvT, where *q* is the number of revolutions or periods of the sensor. When the distance between the displaced sensors is equal to the forward distance traveled during the period *T*, any phase difference applied on to the back sensor will lead to such phase difference when $s_{k}$ reaches the starting position of $s_{0}$, making it easier to configure each sensor. Nevertheless, ignoring or failing to match the distance between sensors with the distance covered by the robot and still trying to reach $T_{x}/s$ can lead to sensors which are not orthogonal to each other, increasing the chance of interference. Hence, if x/v<qT/s, we must increase the distance between sensors or move slower than *v*. On the other hand, if x/v>qT/s we must move faster or decrease the distance between sensors. Still, depending on x/v, this modifications may lead to infeasible speed or unreasonably large distances between sensors.

Another way to adjust phases is to change *T* (and ω), modifying the configuration of the sensor—typically changing the angular step θ. This way, *T* is a variable

$$T=\frac{sx}{qv}$$

which often has limited options which depend on the available configurations of each specific MPS. Still, increasing the angular velocity of the sensor will often impact resolution, since a faster rotation often means larger angular steps. The phase shift approach is therefore highly dependent on phase differences.

### 5.2. Sector Offset

**Figure 8:**
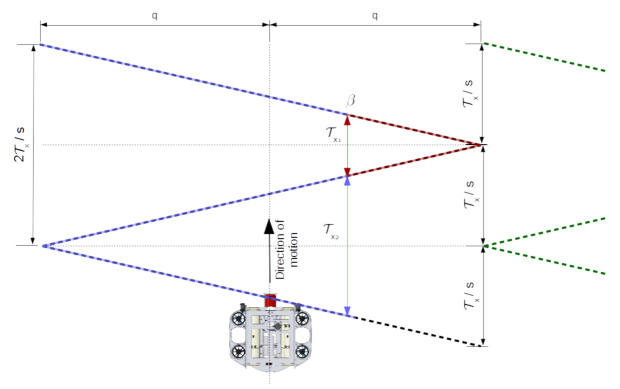
Sector sampling of a flat region. Dashed black lines represent the readings of a sensor, while green dashed lines represent the reading of an adjacent sensor. The distance between readings at the same head position, β, but subsequent time-steps, presents different values—depending on the path taken by the sensor. The Long path (blue dashed) results in greater distance between samples than the shorter path (red dashed). Smaller and larger distances alternate each other and also that the sum of both is $\frac{2T_{x}}{s}$.

In this alternative method of configuring multiple sensors, each sensor is responsible for its own sector. Therefore, it takes T/s seconds to scan its own sector. As each sensor does its sector in parallel, it may look like we can get *s* times faster sampling and *s* times smaller distance between samples than that with a single sensor. However, when the movement from one end of the sector to the other is finished, the head is not back at the start position, as it would be in a full revolution. Therefore, the sensor will have to travel back for another T/s seconds to reach the starting angular position (see Figure 8). In this case, the period of the sensor is 2T/s. Another problem is that the time to reach the same head position to find $T_{x}$ varies depending on the position and direction of movement of the head.

As a result, the distance between samples at the same head position but subsequent periods are no longer constant. In fact, at each end of the interval, the distance between samples is constant and equal to $2T_{x}/s$, which may be assisted by a sample from another sensor between them (see where dashed lines in green connect in Figure 8), which leads to a distance between samples of $T_{x}/s$. Also, if the head is positioned at the middle of the sector, subsequent readings at this position are always equal to $T_{x}/s$. Unfortunately, for the other samples in the interval, the distance decreases and increases linearly depending on the angular position and direction of rotation of the head—clockwise or counter-clockwise. These samples present two possible values: a small value, $T_{x1}$, where the path from the head position to the edge of the sector is such that $0<T_{x1}<T_{x}/s$; and a large value, $T_{x2}$, when the edge of the interval where the head is distant from the current head position, where $T_{x}/s<T_{x2}<2T_{x}/s$. Note that

$$T_{x1}+T_{x2}=\frac{2T_{x}}{s}.$$

Besides, this abrupt change in the distance between samples as the robot moves forward leads to poor and good sampling periodically. As a result, when we use *s* sensors to perform sampling over sectors, the sampling distance is often larger than the desired result of $T_{x}/s$ for half the samples periodically. The opposite is also true, that is, for half the sector samples over the tunnel length will present a smaller distance than T/s. One final note on sectors is the area covered during the half of the sector with smaller distances is smaller than the half of the period when samples are larger than T/s, increasing the chance of missing features of the tunnel.

In order to reproduce the triangular-shaped pattern of sensors configured to work in sectors as in Figure 8, we must also consider the delay between the forward sensor and backward sensors, to achieve phase alignment and the displacement for each sector. Such alignment generates triangle wave patterns, which can be reproduced by means of Algorithm 1. However, as the difference of phase between sensors will affect only the end of the sector at one sample, ignoring it in favor of perpendicular sensors, that is, setting the delay to 0, can help decrease inter-sensor interference.
**Algorithm 1:** Computing the angular position of the head of sensor $s_{k}$ for sector-based configuration.
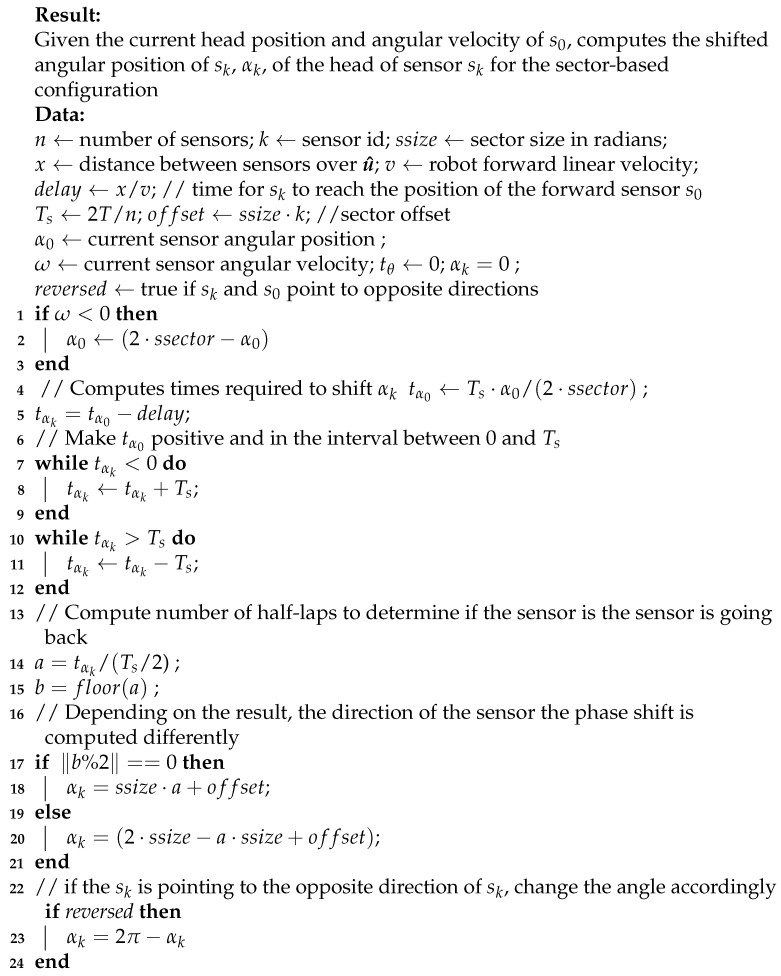


It is worth noting that there is a third way to perform sector sweep using multiple sensors, commanding the head to go back to the initial position once it reaches the end of the sector, and then restart the sweep. However, even for fast sensors the time taken to go back will force distances between samples to be always greater than $T_{x}/s$, impairing sampling quality. Also, even if the time taken to go back to the starting position was 0, a proper alignment of phases would produce results similar to those of the phase shift approach. Therefore, we leave it as an alternative to the reader, but we no longer discuss it in this work.

## 6. Non-Ideal MPSs

When we step from ideal to non-deal MPSs we have to consider the beam shape which, in the case of pen-based sonars, opens in a cone shape with aperture ζ and radius ζ/2. It means the information about sonar range data in one beam is obtained from the projection of such cone on the surface, centered at position α of the head. As we know the size of the angular step, θ, we can estimate the amount of overlap between sensor readings when the robot is stopped at the center of the cross-section as

(22) ϵ=ζ/θ.

Such metric reflects the ratio of overlap between readings. The effect of such an overlap is a smoothing of range data for MPSs. For instance, for the 881L, a commercial MPS, ζ varies with the operation frequency which goes from 600 kHz to 1 MHz, which respectively lead to $\zeta =2.4^{{}^{\circ}}$ for sonars operating at 600 kHz and $1.4^{{}^{\circ}}$ for sonars operating at 1 MHz. Therefore, $1.4^{{}^{\circ}}\leq \zeta \leq 2.4^{{}^{\circ}}$. Table 1 presents the available configuration of angular steps θ, and the overlapping ratio, ϵ for theses two cases.

The ratio ϵ means that a sensor can potentially carry, or compress, range data of many angular steps alone. The larger the ratio, the larger the amount of data a beam carries. Separating such information is not easy if we do not have information about the beam response over the cone or the data we scanning. A traditional probabilistic model [42] can be used to combine sensor readings to update sensor information, but it comes with considerable computational cost when done in 3D. We could also use heuristics considering the pattern of the beam to find points on the surface, for instance using shadows to estimate the height of objects, but it is dependent on the position of the head and also on where on the surface of the tunnel the cone will hit—ceiling, floor or sidewalls. Finally, ray casting and a bilinear interpolation can also be used to obtain estimates of the range data for the entire tunnel.

Features will show up in the map with more or less detail, depending on how we combine data. Little overlap can be good if we have narrow beams and small angular steps, while a wide beam and large angular step leads to poor sampling data. When θ is small and ζ is large, data will probably be smoothed, unless one can decompress beam information. Large θ and small ζ comes with poor interval between samples, but points have a greater chance to be in the correct position in space, but the chance to combine data from several sensors decrease as subsequent beams do not carry information from one another. This can be a problem since some degree of overlap can be used to filter out noisy data. Further, the beam range information comes from an area, instead of a point. Thus, a feature on the surface of the tunnel, which is catch by the cone will be present in the range data, while point-wise sensors can easily miss data. Still, even with the beam, information can be lost—for example, when $theta=2.4^{{}^{\circ}}$ and the sensor operates at 1 MHz, which has no overlap between subsequent rays.

Furthermore, sonars often make discrete measurements over the range of the sensor. The number of range samples can be constant or vary with range, but it always equal to an integer number, *g*. The ratio η=(range_max−range_min)/g defines the smallest measured range value the sensor is able to achieve, given the range. In the equation above, range_min and range_max are the maximum and minimum ranges which can be detected by the sensor. For instance, when the 881L sensor is configured with the range of 10 m, η≈0.01 m.

Finally, the quality of the entire system is defined by many variables. We can control some of them using the robot speed and the configuration of the sensor. Overall, the range of the sensor, the size of T and related values, ζ, ϵ and η together define the quality of sampling with single beam sensors. One important aspect from the present study is the resolution of the sensor does not reflect the distance between samples in the map—as we have seen with T, $T_{x}$, and $T_{y}$.

## 7. Materials and Methods

### 7.1. Sensor

The present paper performs experiments using the specification available for commercially available mechanical profiling sonar with a single ray. We have chosen the Imagenex 881L profiling sonar (Information available at https://imagenex.com/products/881l-profiling (accessed on 20 February 2021)) for our experiments. As any sonar, its response time is dependent on range. However, it has five available configurations which change the revolution period *T* and the size of the angular step, θ—the five configurations are named SLOW, MEDIUM, FAST, FASTER, and FASTEST in the experiments. In addition, its operation frequency can be configured from 600 to 1000 kHz. The sensor is also able to perform both phase shift and sector offset approaches. Table 2 displays the information for each configuration considering the range of 10 m, which is the range set for all experiments.

As we can see from Table 2, the decrease of the angular sampling resolution implies an increase in the revolution period *T*. Also, even though the sampling frequency is high for small θ, it may be misleading, since the revolution will take longer to complete.

### 7.2. Simulations

**Figure 9:**
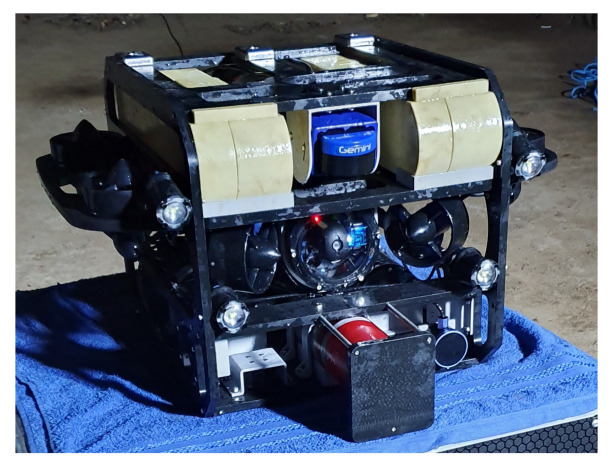
Robot connected to one mechanical profiling sonar (MPS) with the sensor rotation axis aligned to the robot heading.

We made use of a simulation environment which makes use of ROS [36], Gazebo [37], and the uuv_simulator package [41]. We designed two robots for the simulations: VITA 1 (shown in Figure 9) and VITA 2 (currently under construction). The relevant differences between them, for the current series of experiments, were size and payload capacity. VITA 2 is larger than VITA 1 and is designed to operate with up to four MPS Imagenex 881L. We made use of VITA 2 to perform tests with more than one sensor. When required, the 3D reconstruction process was performed using the Poisson reconstruction [43] algorithm, which is one of the algorithms that shows good mapping results [44], depending on the configuration.

### 7.3. Simulated and Real World Environment

**Figure 10:**
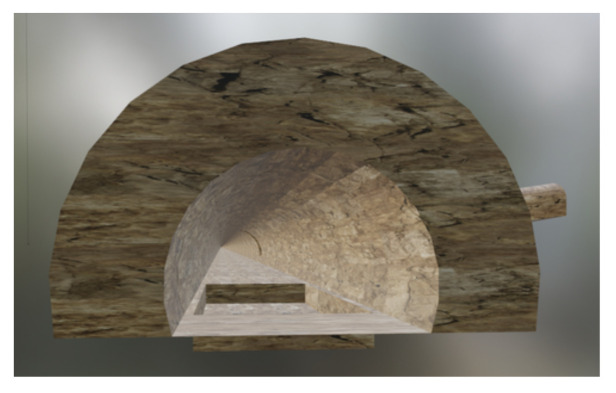
The tunnel scenario considered for simulations and real world tests. The rock trap (the ditch shown in the figure) along with an auxiliary tunnel entrance.

We created a simplified mesh of an actual tunnel from Ceran, a company from the south region of Brazil. The tunnel has a base of 10.4 m, ceiling and side walls comprise a trimmed circle with radius 6.05 m, and its length is around 7 km. Still, in our experiments we concentrated our tests on a section of around 130 m at one end of the tunnel, between the rock trap and an entrance of an auxiliary tunnel connected to the main tunnel (see Figure 10).

## 8. Experiments

In this section we analyzed the metrics from Section 3, considering UUVs equipped with up to four sensors in ideal conditions. A comparison of sector offset and phase shifted sampling is presented in Section 8.1, Section 8.2 and Section 8.3 with up to four sensors. We validated the methodology for the alignment of sensors, showing the effect of phase alignment when sensors are distant from each other, and also the problem of occlusions when sensors are side by side.

**Figure 11:**
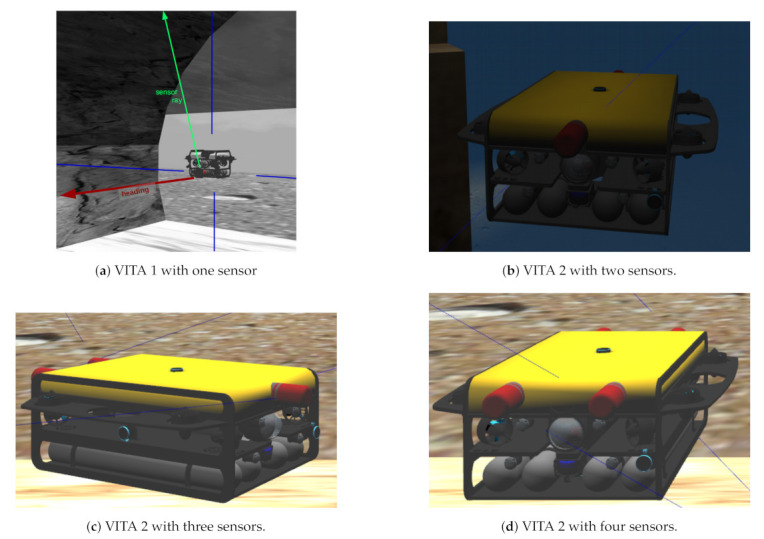
Robots positioned in the entrance of the tunnel. In (**a**), the vector in red depicts the robot heading vector, while the green vector is the ray of the sensor which is always co-planar with the cross-section of the tunnel. Blue lines depict the ping rays of four ping sensors which are used to keep the two robots centered at the cross-section of the tunnel as they move. In (**b**–**d**) we see VITA 2 with several 881L configurations.

We made use of VITA 1 equipped with one 881L sensor positioned at the front of the robot, 0.15 m below the center of mass of the robot Figure 11a. Experiments with two to four sensors were performed with VITA 2 (see Figure 11b–d), where sensors were positioned as follows: when using two aligned sensors, one sensor was positioned at the rear and one at the front of the robot with opposite headings; in the case of 3 sensors, one sensor was centered at the front of the robot and two other sensors were positioned side-by-side and separated by 0.3 m at the back, both with opposite headings with respect to the sensor at the front; and, finally, the configuration with four sensors, had two side-by-side sensors at the back and two side-by-side sensors at the front, also separated by 0.3 m and the sensors at the back having opposite headings with respect to the sensors of the front of the robot. The distance between forward and backward sensors in VITA 2 is 1.102 m. Simulated tests were performed using Gazebo and the uuv_simulator. All robots were equipped with four Bluerobotics pings to give the pilot information about the UUV distance from sidewalls, the ceiling and the floor. All tests were performed with the pilot controlling the robot with a joystick. During the simulations, the robot moved at approximately 0.197 m/s.

### 8.1. Metrics

In this section we present the tests in simulation scenarios for one and then multiple sensors. Table 3 compares experimental and predicted data for minima, maxima, and mean. We also include the standard deviation for analysis of the behavior of the mean distance between subsequent samples, even though the distribution is not a Gaussian. Table 3 shows predictions seem to match experimental data, even though the sample size is too small for statistical analysis. However, we consider the multi-sensor approaches, the Wilcoxon Signed-Rank test comparing the values of estimated and obtained experimentally for all configuration of sensors for $T_{x}$, ${\bar{T}}_{y}$, $T_{y_{min}}$, $T_{y_{max}}$ with significance α=0.01, confirm the use of such parameters as reference for estimating mapping resolution. Using the same approach for sector offset mode, leads to the same results.

Due to the shape of the tunnel we have chosen to use $T_{y1}$ as $T_{y_{min}}$, since the ceiling does not follow the shape of the tunnel, we have studied. We ignore the region of the rock trap and the auxiliary tunnel, giving preference to the regular shape of the main tunnel, which is the use case we are interested in. Also, when we consider ${\bar{T}}_{y}$, $T_{y_{min}}$, $T_{y_{max}}$, and also with $\bar{T}$, $T_{min}$, $T_{max}$, we ignore the effect of motion during the time taken between subsequent angular steps, which results in very small distances traveled, since the robot will move slowly in our experiments—0.197 m/s. For the sector offset approach we ignore valleys in the distance between subsequent samples next to the borders of the sector, since the sensor is decreasing speed due to the control system of the head, which leads to false minima. Such values are discarded since they do not represent our model or the behavior of typical MPSs which steps at every angular position. For $T_{y_{max}}$, we consider the pilot can drift 1.5 m vertically and horizontally. Besides, we consider the robot heading can drift 15 degrees from the direction along the length of the tunnel, ($\hat{u}$). We also include the displacement of sensors vertically and horizontally to compute maxima and minima, summing the robot drift and the sensor displacement with respect to the center of the cross-section of the tunnel, for each of the configuration of sensors and robots.

Table 4 compares data for multiple sensor configurations. For phase shift, we use $T_{x}/s$, while for sector offset we use $2T_{x}/s$. Comparing the two multi-sensor approaches one against the other, results in smaller $T_{y_{max}}$ smaller for sector offset than phase shift approach. Such difference shows specially for three and four sensors. The key point is sensors in sector offset configurations always point outward, as is the case for 3 and 4 881Ls, which have side-by-side sensors separated by 0.3 m. As such, those sensors always point to the closest walls, 0.15 m forward, while continuous spinning will have to scan the opposite and distant side. While this leads to values which tend to benefit maxima measurements when using sector offset for 3 or 4 sensors, such difference vanishes for 2 sensors, since it has zero horizontal offset between sensors and the center of mass of the robot. The Wilcoxon Signed-Rank test comparison leads to no statistical difference between the two approaches with respect to maxima. Still, when we isolate data for three and four sensors the test rejects the alternative hypothesis, that is, that phase shift presents greater values for $T_{y_{max}}$ than sector offset, with significance α=0.05 with a small margin. Also, FASTER and the FASTEST configurations have large differences at the corners, as predicted.

Still, the major difference between the approaches occurs in the maximum interval between samples along the tunnel length, that is, $T_{x}$, which presents significantly higher values for the maxima for the sector offset approach with significance α=0.01. While this is true for half the sector, it can lead to missing features during the tunnel inspection. When we observe the triangular wave pattern, we can see the cost of decreasing $T_{x}$ half the sector is a reduced area covered in such region, while the area covered during large $T_{x}$ values is increased. We draw attention to the fact that once the sensors do not help each other in any multi-sensor configuration with respect to the cross-section, we will have large differences between samples near the side walls and such differences can be larger than the forward gap between sensors, $T_{x}$.

When we compare the predictions for the cross-sections combined with the measurements along the length of the tunnel (see Table 5), we can see that the distance between samples increases. Moreover, the phase shift approach presents consistently greater distances between samples when compared to sector offset. Still, for slow configurations of the sensors, $T_{x}$ is the major cause of large spaces between samples. In fact, T and related metrics can be seen as a sampling interval. Therefore, in order to guarantee the detection of sensors using a multi-sensor strategy, features will only be detected if their size is greater than T. Therefore, in the best case, when using four sensors and traveling with the given forward robot speed leads to the detection of features with size around 0.6 m, which can be decreased if the robot travels slower than 0.197 m/s, considering ideal sensors.

### 8.2. The Impact of Alignment on Tunnel Sampling

**Figure 12:**
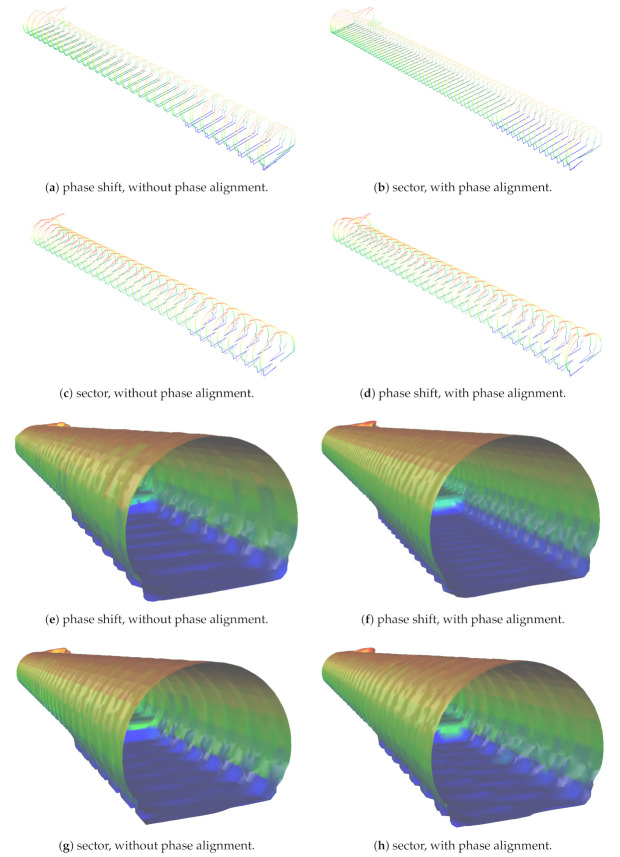
Comparison of the two configuration of sensors with respect to phase alignment.

Here, we compare the phase shift and sector offset configurations in simulated scenarios using the point clouds and meshes for each strategy considering a sensor with a 10 m range. The distance between the forward and backward sensor heads is 1.102 m and VITA 2 is moving at 0.197 m/s. Our test scenario consists of a 130 m slice of a hydroelectric power plant tunnel, next to the rock trap. In order to better illustrate the effect of phase alignment, we select the 881L SLOW configuration setting with two sensors. This way, the distance between samples over then length of the tunnel is more clearly visible than with more sensors and superior sampling results. Figure 12, presents the point clouds of a two-sensor array using phase shift without and with alignment (see Figure 12a,d). Figure 12 presents the same scenario for sector offset. In addition, Figure 12e–h shows the Poisson reconstruction with 8 levels, 1 point per sample and a point weight of 5.

Figure 12e,f highlights the influence of correct phase in the phase shift approach, while the same is not true for sector offset, which presents inferior results but does not seem to suffer influence when ignoring phase alignment (see Figure 12g,h). We compare the two approaches considering aligned phases, with the reference mesh in the interval between the rock trap and the auxiliary tunnel, the unsigned mean distance between the reference mesh and the reconstructed using aligned phase shift is 0.040 m with standard deviation of 0.060 m, while for sector offset the mean distance is 0.057 m with standard deviation of 0.077 m. The unaligned version of phase shift gives us a distance from the reference mesh of 0.050 m of mean and 0.083 m of standard deviation, which is interesting, keeping alignment seems to bring harm to the reconstruction process and a random position of the sensor seems to have provided superior results. The same is not true for phase shift which presents 0.059 m as the mean distance and standard deviation of 0.083 m.

In summary, the phase of sensor can importantly affect the distance between samples of phase shift configurations. Still, when configuring sensors in sectors, there seems to be a negative effect to try to keep interlaced triangle patterns of sectors in terms of 3D reconstruction. The unaligned results of phase-shift are inferior to those with sectors—be it aligned or unaligned. However the maxima of distances along the length of the tunnel, which reflect the distance between consecutive scans of cross-sections is visibly smaller in the point clouds of aligned phase shift (see Figure 12b) than the point cloud of the sector offset approach (see Figure 12d). We highlight it is just a mere difference in configuration pattern, not the amount of points in any of the case studies above.

### 8.3. Three and Four Sensors—The Problem of Occlusion in Phase Shift

While the sector off-set approach will not suffer occlusion problems, this is not the case for the phase shift approach. In fact, phase-shift will suffer angular occlusions every time sensors are side by side—that is, occlusions will only occur when using the three and four sensors layout.

**Figure 13:**
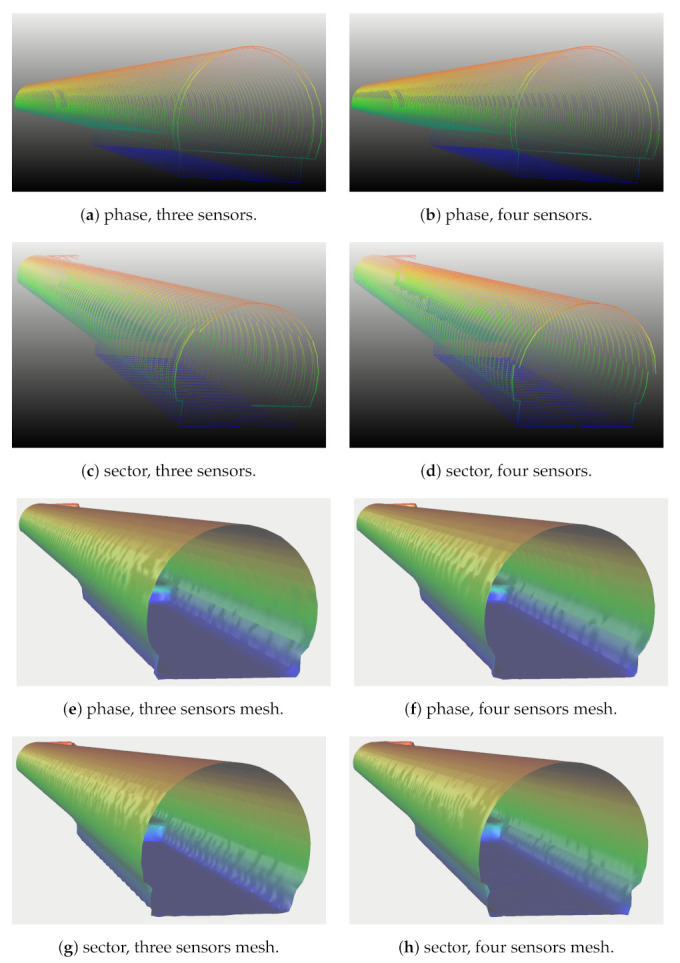
The problem with occlusions in the phase shift approach is shown in (**a**,**b**) when using sensors side by side, for the MEDIUM configuration of the 881L sensor, which makes it easier to see the resulting sampling of both approaches. Note the reading gaps at the side walls. The space between sensors also results in gaps in the sector approach, the gap is smaller on side walls than the phase shift approach, see (**c**,**d**). Comparing figure (**a**) with (**c**) and figure (**b**) with (**d**), the resolution difference between the two approaches. The resulting meshes for the phase shift and sector approaches for three and four sensors are shown in (**e**,**f**) and (**g**,**h**), respectively. The phase shift approach for three and four sensors shows aliasing at the side walls at the occlusions, but there is little aliasing at the floor for three and four sensors. Note the aliasing in (**g**), for sector offset for three sensors shown at the regions in blue next to the floor, while the mesh reconstruction algorithm can decrease the aliasing when using four sensors in (**h**). Also aliasing at side walls seems to be smaller for the sector offset than the phase shift approach.

Moreover, the occlusion angle is a function of the tangent of the distance between sensors, which we set to 0.3 m, and the dimensions of the opposite sensor—that is, the radius of the cylinder of the 881L sensor, which is around 0.04 m. The result of such occlusions is a gap in readings which will show up as missing samples in the side walls (see Figure 13), which will generate a spatial gap of approx. 1.7 m and an occlusion angle of around 15.07 degrees. Therefore, occlusions in the phase shift case lead to under sampling at the side walls, dependent on the number of sensors and how the phase of sensors is configured.

For three sensors, Figure 13a, the backward sensors occlude one another, leading to twice the distance between samples along the length of the tunnel when the gap is present, decreasing the quality of resolution at occluded regions. The four sensors’ layout suffers from such problems twice, at forward and backward side by side sensor pairs. The behavior of the resolution is dependent on how the phase of sensors is set. If the phase between side by side sensors is 180 degrees, the distance between samples along the length of the tunnel is doubled at occluded regions due to missing data. We do not use this strategy because sensors will always face one another, increasing the chance of interference between sensors. However, if the phase between side by side sensors is 90 degrees (see Figure 13b), this will lead to two subsequent sensor gaps, making the distance between samples along the length of the tunnel suffer an increase of four times periodically at side walls.

We proceed with the comparison of the phase shift and the sector approach with the reference mesh, as we did before for two sensors. The sector offset approach for three sensors presents a mean distance of 0.037 m, while the standard deviation is 0.106 m, while the phase shift approach for three sensors presents a mean distance of 0.018 m with a standard deviation of 0.031 m. The same result for four sensors in the sector offset reveals a mean distance from the reference mesh of 0.02 m, and standard deviation of 0.036 m. while the phase shift approach presents a mean distance of 0.016 m and standard deviation of 0.025 m. That is, even with the occlusions of three and four sensors results with the phase shift approach are slightly superior in terms of mesh reconstruction. Still, the four sensors approach using either phase shift or sector offset has an additional gap when compared to three sensors. In the former case the gap is not continuous and caused by occlusions but larger in terms of area, while in the latter case the gap is continuous but smaller, caused by the distance separating the two forward sensors.

While the area of occlusion of phase the shift approach is larger than the space between sensors gap created in the sector offset, spreading to an entire angle of the field of view, the occluded area is not continuous, which is beneficial for the mesh reconstruction algorithm, see Figure 13e–h. This summed with its smaller distance between samples in the remaining areas gives a small advantage to the phase shift approach even for configurations with occlusions. The advantage of the phase shift approach lies on the resolution outside occluded areas, but if these areas are important it can be a limitation of the strategy and sector offset is a viable option. The sector approach can also be improved for tunnels if we place sensors one above the other and we can place them closer to each other, to give preference to ceiling and floor, leaving gaps at side walls. The phase shift approach improves, if we separate the sensors to decrease the size of occluded areas.

## 9. Real World Experiments

We could perform experiments with VITA 1 in the SLOW configuration inside the actual tunnel. We use it to validate the tests with our first UUV prototype. VITA 2 is still under construction, which is why no tests with it using more than one sensor will be provided.

### 9.1. The Power Plant Environment

In this section, we report the general conditions of the environment VITA 1 has faced.

The underwater tunnel was under maintenance, and there was a leak which resulted in a counter or favorable current around 0.10 m/s, depending on the UUV direction—upstream or downstream. The robot was able to move inside the tunnel despite such current.

The visibility inside the tunnel was between one and three meters, which is a common trait of river waters [2]. We have used a Gemini 720im sonar to navigate inside the tunnel and the 881L for manual navigation. Instead of a black or a Secchi disk, we performed a custom test. Such test consisted of submerging VITA 1 with its four led lights on maximum power, and measure the depth where the vehicle vanishes completely from sight using our depth sensor. We performed such test twice which resulted in a visibility of around three meters next to the power house. Logistics at the water intake made a similar test a bit more difficult, but divers reported visibility between 0.5 and 1 m, probably due to the large number of debris from the river, wood, and other objects.

**Figure 14:**
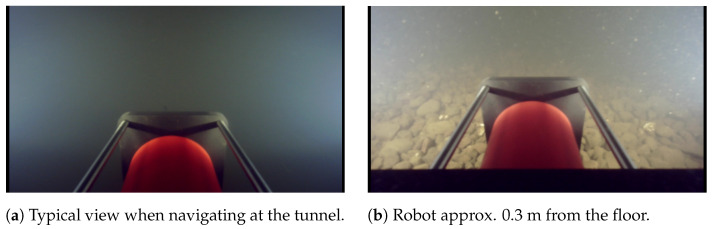
Low visibility using the low light camera at the tunnel: centered at the cross-section (**a**) and close the floor (**b**).

When navigating inside the tunnel, we performed manoeuvres placing the robot at the center of the tunnel cross-section, and every time, it was not possible to see the side walls, even with the robot facing them. The typical video image is shown in Figure 14a, which we have hours of video recording with very similar images. An image when the UUV is less than 0.5 m from the floor is shown in Figure 14b. As one can see, the visibility is low even in a region next to the rock trap which usually has mostly stones, with the UUV almost touching the floor.

### 9.2. Mapping

In order to construct the map, we used telemetry data from the IMU with a compass to obtain an estimate of the pose of the robot inside the tunnel. We used information from the pressure sensor, a Bar100 high-resolution 1000 m Depth/Pressure sensor, to obtain depth information and a tether counter to obtain an estimate of the robot position along the tunnel length. Finally, as we did in the simulated experiments, we also used four Bluerobotics Ping Echosounders pointing to vertical and horizontal walls to obtain an estimate of the robot position along the plane of the cross-section of the tunnel. No SLAM technique was used at this time, beyond the correction of the position of the robot using ping data and the known information that the tunnel is straight to position the robot inside the tunnel. That is, we used the differences between left and right pings, to detect motion to the left or to the right and top and bottom pings to keep the robot away from the walls, however depth was controlled by the depth sensor at this time. The UUV was manually controlled in this experiment making use of a Teledyne Gemini 720im imaging sonar to help guiding the pilot during the operation. Imaging sonar data was not used as input for mapping due to noise. It is worth noting that the simulated tunnel was a simplified version of the actual tunnel at Ceran, a utilities company from the south region of Brazil. Furthermore, the 881L sensor was configured with the range of 10 m, operating at a frequency of 1 MHz.

**Figure 15:**
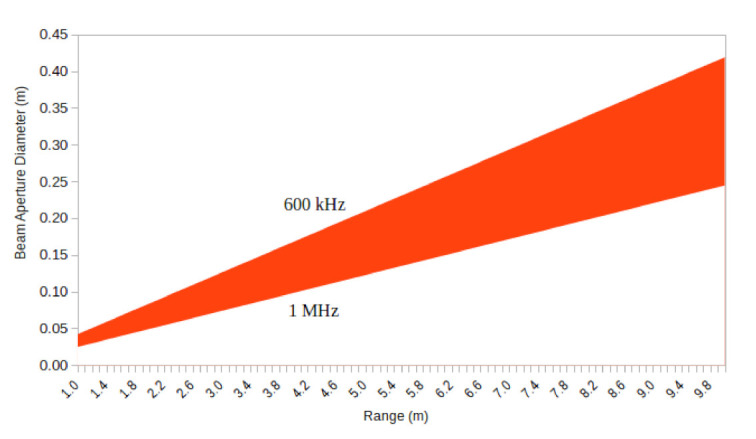
The behavior of projected distances on a surface as the range and frequency change.

It is important to note that the 881L is not an ideal sensor, as we have been working with until now. In fact, its beam aperture diameter widens with the increase of the frequency of operation, going from $1.4^{{}^{\circ}}$ to $2.4^{{}^{\circ}}$ which leads to projections of the cone that vary with range—see Figure 15. In fact, we can see that the beam is indeed wide reaching up to around 25 cm, with its projection increasing as it moves towards the corner. Besides, the overlap between the sonar at different head positions will occur with increasing overlap as the angular step gets smaller. Such behavior is similar to $T_{y_{m}ax}$—that is, the projection of the beam shape follows the tangent function as well. This is expected behavior from the sensor but it will often lead to smoothing of features when there is two much overlapping between sensor readings. In addition, 881L also has a resolution of 500 points per scan, which for ten meters leads to approximately 0.02 m of sensor resolution.

**Figure 16:**
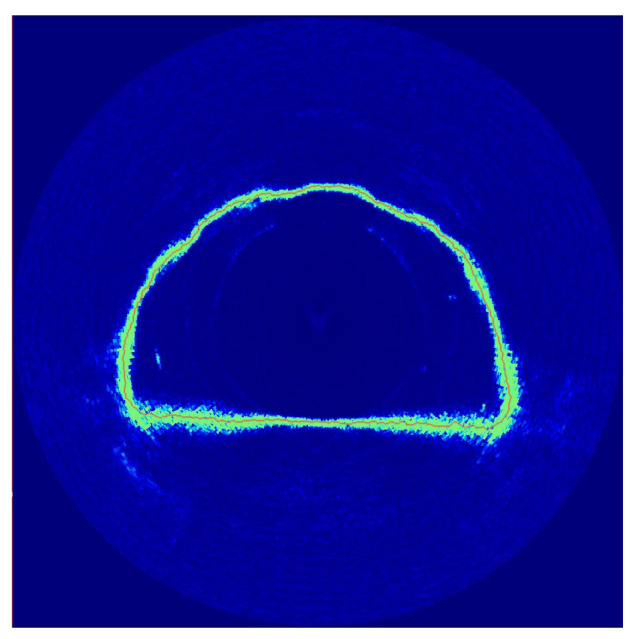
Cross-section of the tunnel and the red thinning line which represents its skeletonization followed by pruning.

First, we stop the robot in the center of the cross-section to measure the perimeter of the tunnel, considering the effect of the beam aperture to check if we can obtain an estimate of the mean distance between samples at the cross-section in real world conditions. We use bilinear interpolation of range values of the 881L sensor to determine where the tunnel surface is. The profile of the tunnel is shown in Figure 16. We perform thinning, pruning, and then manual cleaning to ensure eight connectivity of the tunnel profile. We opted for such an approach since the thickness of the tunnel profile make it difficult to know where the floor or the ceiling are. The skeletonization process leads to an average of the estimate of the perimeter of the cross-section, which results in 33.09 m, versus the nominal blueprint of the plant of 34.17 m, which is in accordance with what we expect our from simulations. One may also notice the group of readings near the surface next to the corners thickens. This is a combination of the increase in the distance between samples and the beam aperture, which reduces the amount of information we can obtain from such regions, confirming our expectations. That is, when we interpolate non-ideal cone shaped readings the result will degrade the precision of the sensor and lower its accuracy. This is a limitation of the approach.

**Figure 17:**
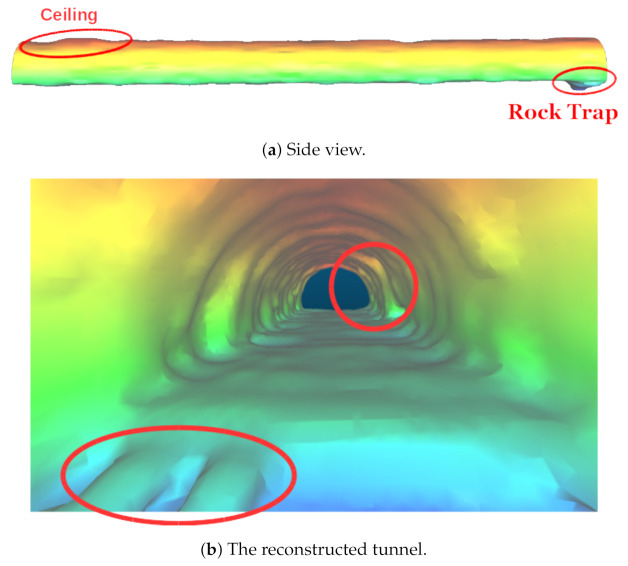
The reconstructed tunnel. Regions of interest are marked in red. Note we can detect height differences in the ceiling and also some details of the end of the rock trap. Due to poor sampling along the tunnel length we cannot get a lot of details of the side tunnel we saw in previous experiments.

Regarding $T_{x}$, it depends on the forward speed which was measured with the tether counter. The estimated mean forward velocity of the UUV was 0.197 m/s, which will lead to mean values of $T_{x}=4.255$. Still, manual coordination between tether operator and pilot can be hard to achieve, especially when both are distant from one another. In respect of inspection, this is poor space between samples, but it was still used to show we can reconstruct the tunnel with the initial prototype. We present the part of the tunnel which we have reconstructed with VITA 1 in our first visit to the tunnel in Figure 17.

## 10. Discussion

Experiments with simulated scenarios have shown that we can predict the distance between samples including maxima and minima. The main problem for sensors which have a slow angular velocity or pooling rate is the distance between consecutive profiles of the cross-section of the tunnel. Even if we work with ideal sensors, the distance between samples can be large. Moving away from the center of the cross-section results in important changes in the distance between samples, affecting maxima and minima of the distance between samples. Therefore, it is important that the control system of the robot to be robust, keeping it at the center of the cross-section as it moves, or the input data for mapping will not be optimal.

Regarding the sensor model, which we can use to combine all readings when the sensors are not ideal, a few considerations can be made. First, we could use a traditional probabilistic model to update sensor information, but it comes with high computational costs when done in 3D. We could also take advantage of the aperture of the sonar ray over nearly flat regions, and use the upper and lower angular limits of the beam to point the exact position of the beam in these regions. However, it demands some of inference to know where such regions are. Finally, as we are using an MPS, such points will not lead to continuous sweep as an MBS performing bathymetry, since the pattern of the point cloud with MPSs will be spiraled. It may lead to a gap between points which might never be filled. However, these ideas need further experiments and validation.

The positioning of sensors can help increase or decrease the distance between samples, but it can also leave holes in the tunnel profile caused by the space between sensors or even lead to sensors which can block each other. One result of the experiment is that even if we combine sensors at different positions, they will probably need adjustments of phase in order to keep a uniform space between samples along the length of the tunnel, which is particularly important if we are going to keep the sensors spinning continuously. The rule after phase adjustment was uniform space between samples but sensors which are not orthogonal to each other. Experiments have shown that in multi-sensor configurations, at first we have to choose a forward speed with which the robot will operate inside the tunnel. Then, we define the distance between sensors, to simplify phase adjustment. While the sector offset approach has shown inferior sampling results, it might be easier to configure since it is less sensitive to phase shifts. In other words, we ignore the phase in favor of the orthogonality of sensors, which can help minimizing interference.

Interference is a problem which we are currently working on. Sensors operating in different frequencies have a smaller of inter-sensor interference than when all of them operate on the same frequency—a feature of 881L. Still, some sensors may not have such feature. Also, the beam shape may change as the operating frequency changes, thickening the ray and decreasing sampling quality for those sensors operating with frequencies smaller than 1 MHz. Unexpected side lobes in the beam shape can make the process hard to solve. Using barriers to separate the beams is also under consideration, but it may not be very effective due to multi-path reflections of the rays of all sonars on the tunnel walls.

If water visibility inside the tunnel were better or the tunnel smaller, vision based sensors could have been used, improving the resolution and precision of the entire system. However, during an entire week the visibility at the tunnel remained poor. Besides, the other tunnels we are addressing in the future are wider than the one we present in the paper, where the range of the sensor will most likely increase, surpassing the maximum range of commercial visual based sensors. Having said that, we believe not all rivers and tunnels offer the same conditions than the one we have tested—the reader will have to test such conditions on site to decide the feasibility of use of vision based sensors. As we have shown in the formalization, the use of MPSs with good resolution can lead to considerably slow forward speeds, due to the low refresh rate of the sensor, and longer duration of missions, specially if the tunnel is long. Breaking the tunnel into parts can be an option if the duration of the mission is impractical.

## 11. Conclusions

In this paper we present a clear way to analytically determine the resolution of a system equipped with a set of MPSs when they are used in tandem for mapping tunnels. The system is tested in simulation using Gazebo, ROS, and the uuv_simulator. We have prepared two robots, one with a single sensor and the other which can simulate different configurations and quantities of sensors. For this purpose we have used the Imagenex 881L sensor data as reference, even though our work can be extended to other sensors, including multi-beam sensors.

We demonstrate that sampling with an MPS or multiple MPSs resides in two important metrics: the distance covered by the robot at each revolution of the system; and the angular step size of the sensor. We also show that the distance covered by the robot at each revolution often is larger for UUVs operating in tunnel scenarios, mainly due to the typically slow angular velocity of MPSs. As such, we may have to move slower, depending on the desired resolution, to guarantee the distance between samples remains small. In other words, with such a solution we trade quality for mapping speed. Special attention must be paid in large tunnels, since time can be critical for the operation, occurring during the stops of hydroelectric power plants for maintenance. Also, we highlight the importance of knowing the speed of the robot inside tunnels, where a DVL or a tether counter can be valuable—specially if the tunnel does not have features which can be used to estimate the speed and position of the robot along the tunnel length. Overall, results show that engineers and technicians must be aware of the quality of sampling the system is capable to achieve during such tunnel inspections.

Regarding the sampling quality of the system, we mathematically show that when using MPSs the distance between samples on the floor next to the side walls usually present the largest distance between samples for the entire tunnel. Also, the floor is where debris from side walls and ceiling often stop and where valuable information must be collected. Furthermore, the largest range measured by a robot centered in a cross-section of the tunnel is often at the corners—which can be used as a reference to estimate the operation range of the sensor. Therefore, the proper definition of the maximum distance between samples on the floor is critical for inspection, and the worst case scenario can be used as reference parameter to determine the resolution quality inside tunnels.

We have also compared two different approaches for the combination of sensors focusing the mapping of tunnels: continuous phase shift rotation and sector offset segmentation of the cross-section of the tunnel. We show that the phase shift configuration presents uniform sampling over the tunnel length, while sector sampling does not. In the worst case scenario, the sampling distance over the tunnel length for sector offset is twice the size of the worst case scenario of the phase shifted configuration. On the other hand, since sampling of each sector is carried out by one sensor, it is less prone to sensor synchronization problems than the phase-shift configuration. An important restriction of the phase shift method is self occlusion of side by side sensors, which can lead to areas where the resolution can be as poor as using a single sensor, which does not occur with the sector offset approach. If occluded areas are not an option, sector offset can be used, but it will present inferior resolution elsewhere.

Finally, we have also presented a first map of part of an actual tunnel, where even with a slow sensor configuration we could detect some interesting features of the tunnel. We report the visibility at our testing site, which is between 0.5 and 3 m, with poor visibility at the water intake and a better visibility at next to the power house. Still, we are currently finishing the construction of our second prototype which can hold up to four MPSs—starting with two sensors for testing purposes. An important limitation of the present work is the absence of tests with multiple sensors in real world scenarios, which we plan to address in the future once the second prototype is ready, addressing frequency interference and reflections. We are planning to test the new prototype in two power plant tunnels, as soon as it is feasible, given the logistics for 2021. We are also working on a better localization strategy to minimize localization errors. Another area we are working on is control, which is now being implemented in the robot to ensure a better motion model.

## Figures and Tables

**Table 1:** Superposition of rays considering different sensor configurations and frequencies.

θ	$0.3^{{}^{\circ}}$	$0.6^{{}^{\circ}}$	$0.9^{{}^{\circ}}$	$1.2^{{}^{\circ}}$	$2.4^{{}^{\circ}}$
ϵ @600 kHz	8	4	2.67	2	1
ϵ @1 MHz	4.67	2.33	1.55	1.16	0

**Table 2:** Imagenex Parameters for the Range of 10 m.

Parameter	SLOW	MEDIUM	FAST	FASTER	FASTEST
θ (degrees)	$0.3^{{}^{\circ}}$	$0.6^{{}^{\circ}}$	0.9	$1.2^{{}^{\circ}}$	$2.4^{{}^{\circ}}$
samples/rev	1200	600	400	300	150
*T* (s)	21.6	10.8	8	6.6	4.8
$f_{s}$ (samples/s)	55.55	55.55	50	45.45	31.25

**Table 3:** VITA1 with one 881L sensor and range of 10 m: $T_{y}$ = sampling dist. along the tunnel cross section; ${\bar{T}}_{y}$ = mean; $T_{y_{min}}$ = min.; $T_{y_{max}}$ = max.; $T_{x}$ = dist. between samples along the tunnel length.

881L Mode	Experimental Data (m)					Baseline (m)			
	$T_{x}$	${\bar{T}}_{y}$	${\bar{T}}_{y}$ std	$T_{y_{\min}}$	$T_{y_{\max}}$	$T_{x}$	${\bar{T}}_{y}$	$T_{y_{\min}}$	$T_{y_{\max}}$
**SLOW**	4.200	0.032	0.009	0.016	0.099	4.255	0.029	0.013	0.014
**MEDIUM**	2.090	0.058	0.015	0.038	0.156	2.128	0.057	0.026	0.203
**FAST**	1.527	0.086	0.033	0.045	0.337	1.573	0.086	0.039	0.301
**FASTER**	1.283	0.114	0.049	0.051	0.417	1.300	0.115	0.053	0.396
**FASTEST**	0.947	0.226	0.079	0.075	0.552	0.946	0.230	0.100	0.752

**Table 4:** Experiments for the range of 10 m considering 2, 3 and 4 881L sensors using VITA 2, comparison of Phase-Shift and Sector Offset, where s = nr. of sensors; $T_{x}/s$ = phase shift resolution along the tunnel length; $2T_{x}/s$ = sector offset resolution along the tunnel length. Numbers in bold indicate winning approach.

881L Mode	Sensors	Experimental Data (m)										Baseline (m)				
		Phase Shift					Sector Offset									
		$T_{x}/s$	${\bar{T}}_{y}$	${\bar{T}}_{y}$ std	$T_{y_{\min}}$	$T_{y_{\max}}$	$2T_{x}/s$	${\bar{T}}_{y}$	${\bar{T}}_{y}$ std	$T_{y_{\min}}$	$T_{y_{\max}}$	$T_{x}/s$	$2T_{x}/s$	${\bar{T}}_{y}$	$T_{y_{\min}}$	$T_{y_{\max}}$
**FASTEST**	**2**	**0.470**	0.226	0.083	**0.074**	**0.542**	0.939	**0.224**	0.082	0.077	0.575	0.470	0.941	0.230	0.100	0.745
**FASTER**		**0.647**	0.114	0.051	**0.052**	**0.414**	1.300	**0.113**	**0.050**	0.056	0.418	0.647	1.294	0.115	0.05	0.391
**FAST**		**0.788**	0.086	0.034	**0.046**	**0.345**	1.572	0.086	0.034	0.049	0.362	0.784	1.568	0.086	0.041	0.297
**MEDIUM**		**1.056**	0.058	0.016	**0.035**	**0.154**	2.115	0.058	0.016	0.037	0.156	1.058	2.117	0.057	0.027	0.201
**SLOW**		**2.098**	0.032	0.009	0.017	0.104	4.174	0.032	0.009	**0.016**	**0.098**	2.116	4.234	0.029	0.014	0.102
**FASTEST**	**3**	**0.320**	0.226	0.083	**0.072**	0.597	0.635	**0.221**	0.083	0.076	**0.567**	0.314	0.627	0.23	0.109	0.759
**FASTER**		**0.429**	0.114	0.051	0.049	0.419	0.865	**0.112**	**0.050**	**0.048**	**0.405**	0.431	0.862	0.115	0.055	0.399
**FAST**		**0.525**	0.086	0.035	**0.040**	0.364	1.003	**0.085**	**0.034**	0.046	**0.335**	0.523	1.045	0.115	0.055	0.399
**MEDIUM**		**0.709**	0.058	0.016	**0.029**	**0.154**	1.416	0.058	0.016	0.036	0.168	0.706	1.411	0.057	0.027	0.205
**SLOW**		**1.410**	**0.031**	0.009	**0.015**	0.099	2.832	0.032	0.009	0.019	0.099	1.411	2.822	0.029	0.014	0.104
**FASTEST**	**4**	**0.229**	0.226	0.081	0.088	0.585	0.461	**0.217**	0.081	**0.074**	0.585	0.235	0.470	0.23	0.109	0.759
**FASTER**		**0.323**	0.114	0.050	**0.057**	0.491	0.641	**0.111**	**0.049**	0.058	**0.401**	0.323	0.647	0.115	0.055	0.399
**FAST**		**0.394**	0.086	**0.047**	**0.043**	0.359	0.790	**0.084**	0.033	0.044	**0.340**	0.392	0.784	0.115	0.055	0.399
**MEDIUM**		**0.532**	0.058	0.016	0.034	**0.185**	1.066	**0.057**	**0.015**	**0.031**	0.189	0.529	1.058	0.057	0.027	0.205
**SLOW**		**1.056**	0.032	0.010	0.015	0.101	2.100	**0.031**	**0.009**	**0.001**	0.101	1.058	2.117	0.029	0.014	0.104

**Table 5:** Comparing Experimental data for multi-sensor approaches considering $\bar{T},\;T_{min},\;T_{max}$, where T is a combination of $T_{y}$ and $T_{y}$, which varies depending on $T_{y}$.

881L Mode	Sensors	Phase Shift			Sector Offset		
		$\bar{T}$ (m)	$T_{\min}$ (m)	$T_{\max}$ (m)	$\bar{T}$ (m)	$T_{\min}$ (m)	$T_{\max}$ (m)
**FASTEST**	2	0.522	0.476	0.717	0.965	0.942	1.101
**FASTER**	2	0.657	0.649	0.768	1.305	1.301	1.366
**FAST**	2	0.793	0.789	0.860	1.574	1.573	1.613
**MEDIUM**	2	1.058	1.057	1.067	2.116	2.115	2.121
**SLOW**	2	2.098	2.098	2.101	4.174	4.174	4.175
**FASTEST**	3	0.392	0.328	0.677	0.672	0.640	0.851
**FASTER**	3	0.444	0.432	0.600	0.872	0.866	0.955
**FAST**	3	0.532	0.527	0.639	1.007	1.004	1.057
**MEDIUM**	3	0.711	0.710	0.726	1.417	1.416	1.426
**SLOW**	3	1.410	1.410	1.413	2.832	2.832	2.834
**FASTEST**	4	0.322	0.245	0.628	0.510	0.467	0.745
**FASTER**	4	0.343	0.328	0.588	0.651	0.644	0.756
**FAST**	4	0.403	0.396	0.533	0.794	0.791	0.860
**MEDIUM**	4	0.535	0.533	0.563	1.068	1.066	1.083
**SLOW**	4	1.056	1.056	1.061	2.100	2.100	2.102

**Figure A1:**
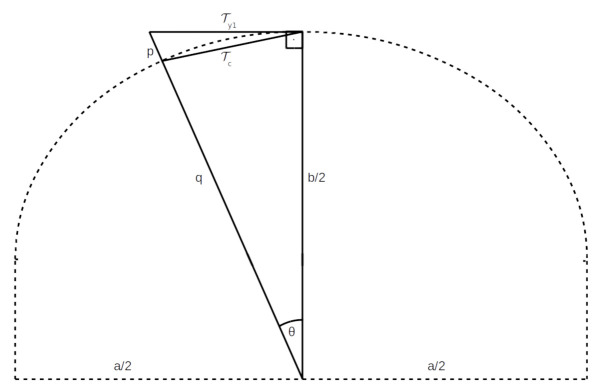
The upper part of a tunnel with an arbitrarily convex but symmetric ceiling. Note that $T_{y1}$ and b/2 always form a square angle, therefore p+q=b/(2cosθ).

## Data Availability

Data is contained within the article.

## Abbreviations

The following abbreviations are used in this manuscript:

3D	Three Dimensional
DVL	Doppler Velocity Log
MBS	Multi-Beam Sonar
MPS	Mechanical Profiling Sonar
SLAM	Simultaneous Localization and Mapping
ROS	Robot Operating System
UUV	Unmanned Underwater Vehicle

## Appendix A

**Proof of Theorem 2.** In order to prove that the spatial distance between consecutive samples increases on the floor, we just need to prove that distance between one step, as the sensor moves *m* steps, is smaller than the distance measured when the sensor moves m+1, that is:

(A1) $$∥\frac{b(\tan(m\theta )-\tan((m-1)\theta )}{2}∥<∥\frac{b(\tan((m+1)\theta )-\tan(m\theta ))}{2}∥.$$

Since we are choosing the step angle to be small where $0^{{}^{\circ}}<m\theta <90^{{}^{\circ}}$. As a consequence $\theta <90^{{}^{\circ}}/m$ and m≥2, leading to tangent values which are always positive and we can remove the modulus sign. The proof for the other quadrant can be derived by using the symmetry of the scenario, which generates mirrored samples on the floor at the opposite quadrant. Therefore, Equation (A1) can be rewritten as:

$$\begin{array}{c} \tan(m\theta )-\tan((m-1)\theta )<\tan((m+1)\theta )-\tan(m\theta )\\ 2\tan(m\theta )<\tan(m\theta +\theta )+\tan(m\theta -\theta )\\ 2\frac{\sin(m\theta )}{\cos(m\theta )}<\frac{\sin(m\theta +\theta )}{\cos(m\theta +\theta )}+\frac{\sin(m\theta -\theta )}{\cos(m\theta -\theta )}\\ \;\\ 2\frac{\sin(m\theta )}{\cos(m\theta )}<\frac{\sin(m\theta +\theta )\cos(m\theta -\theta )+\sin(m\theta -\theta )\cos(m\theta +\theta )}{\cos(m\theta +\theta )\cos(m\theta -\theta )}\\ \;\\ \frac{\sin(m\theta )}{\cos(m\theta )}<\frac{\sin(2m\theta )}{2\cos(m\theta +\theta )\cos(m\theta -\theta )}. \end{array}$$

If on the right side we use the double-angle formula for the sine at the numerator and the product-to-sum of cosines at the denominator, we have:

$$\begin{array}{c} \frac{\sin(m\theta )}{\cos(m\theta )}<\frac{\sin(2m\theta )}{2\cos(m\theta +\theta )\cos(m\theta -\theta )}\\ \;\\ \frac{\sin(m\theta )}{\cos(m\theta )}<\frac{\sin(2m\theta )}{cos(m\theta +\theta +m\theta -\theta )+\cos(m\theta +\theta -(m\theta -\theta ))}\\ \;\\ \frac{\sin(m\theta )}{\cos(m\theta )}<\frac{2\sin(m\theta )\cos(m\theta )}{\cos(2m\theta )+\cos(2\theta )}\\ \;\\ 1<\frac{2{\left(\cos\left(m\theta \right)\right)}^{2}}{2{\left(\cos\left(m\theta \right)\right)}^{2}-1+\cos\left(2\theta \right)} \end{array}$$

$$\begin{array}{c} 2{\left(\cos\left(m\theta \right)\right)}^{2}-1+\cos\left(2\theta \right)<2{\left(\cos\left(m\theta \right)\right)}^{2}\\ \;\\ \cos(2\theta )<1. \end{array}$$

Since $0^{{}^{\circ}}<\theta <90^{{}^{\circ}}/m$ the inequality holds, and the distance between samples increases between every consecutive pair until we reach the corner connecting the floor to the side walls, as expected. As a result, the pair at the corner has the greatest distance between samples on the floor, while the pair of samples when the sensor ray is perpendicular to the floor the smallest. □

## Appendix B

**Proof that $T_{c}<T_{y1}$.** We can show that the ceiling has a smaller distance between samples than the smallest distance we can get on the floor by showing that $T_{c}<T_{y1}$:

$$\begin{array}{c} ∥b\sin(\theta /2)∥<∥b\frac{\tan\theta }{2}∥\\ \;\\ \sin\left(\theta /2\right)<\frac{\sin\theta }{2\cos\theta }\\ \;\\ \sin\left(\theta /2\right)<\frac{2\sin(\theta /2)\cos(\theta /2)}{2\cos\theta }, \end{array}$$

leading to the inequality,

(A2) $$\frac{\cos(\theta /2)}{\cos\theta }>1,$$

which is true for $0<m\theta <90^{{}^{\circ}}$, since the cosine of a positive angle in the first quadrant, when halved, is always greater than the cosine of the angle itself. Therefore, the space between samples on the ceiling is smaller than the smallest space between samples on the floor, making the distance between samples on the ceiling smaller than the best case scenario at the floor, as expected. Furthermore, if the upper part of the tunnel is convex and symmetric, such as Figure A1, we can also show that $T_{c}<T_{y1}$. By using cosine law and making b/2=s to shorten the equations we can proceed to:

$$\begin{array}{c} T_{c}<T_{y1}\\ \;\\ q^{2}+s^{2}-qs\cos\theta <{\left(p+q\right)}^{2}+s^{2}-\left(p+q\right)s\cos\left(\theta \right)\\ \;\\ -p^{2}-2pq<\left(qs-\left(p+q\right)s\right)\cos\theta \\ \;\\ -p(p+2q)<-ps\cos\theta \\ \;\\ (p+2q)>s\cos\theta . \end{array}$$

Since we know p+q=s/cosθ, we have

q+scosθ>scosθq>s(cosθ)2−1cosθq>s(cosθ)2−1−(sinθ)2cosθ

$$\begin{array}{c} q>-s\tan\theta \sin\theta \\ q>-\frac{b}{2}\tan\theta \sin\theta \end{array}$$

As $0<m\theta <90^{{}^{\circ}}$ and q and b are positive, the right side is always negative. Therefore, q is always greater than the left side. Also, as the ceiling is symmetric, $T_{c}<T_{y1}$ as expected. □

## Appendix C

**Proof that ${\bar{T}}_{y_{p}}\leq p/n$.** Let the perimeter of the cross-section of the tunnel be represented as graph G={V,E}, where V is the set of vertices which comprise the cross-section of the tunnel and E are the set of edges connecting them. In this sense, G a cycle where all nodes have degree two—that is, every node x∈V is connected to two other nodes w∈V and z∈V by two edges {w,x} and {x,y} of sizes ∥{w,x}∥ and ∥{x,y}∥. The sum of the size of all edges is the perimeter, *p*, of the cross-section. The path followed from *x* to itself leads to a revisit of all nodes and edges. If this path is split into *n* sub-paths of arbitrary sizes, the sum of the edges in all sub-paths is still the perimeter. However, for every sub-path we have a starting node $a_{i}\in V$ and ending node $b_{i}\in V$. Let us say that the sum of the sizes of all edges between $a_{i}$ and $b_{i}$ is $s_{i}$. If $a_{i}$ and $b_{i}$ are nodes sampled by a sensor, the distance between samples is given by the size of the line segment $b_{i}-a_{i}$ which connect $a_{i}$ to $b_{i}$ and has size $∥b_{i}-a_{i}∥$. If all nodes in the interval delimited by $a_{i}$ and $b_{i}$ belong to the same line, $∥b_{i}-a_{i}∥=s_{i}$, since the smallest distance between two points is a line. However, if the nodes of the sub-path do not form a straight line, $∥b_{i}-a_{i}∥<s_{i}$. Therefore, $∥b_{i}-a_{i}∥\leq s_{i}$ for all sub-paths. Since we know that

$$\sum_{i=0}^{n-1}s_{i}=p,$$

and also that

$$\sum_{i=0}^{n-1}∥b_{i}-a_{i}∥\leq \sum_{i=0}^{n-1}s_{i},$$

we can conclude that

(A3) $$\sum_{i=0}^{n-1}∥b_{i}-a_{i}∥\leq p.$$

Thus, the mean distance between samples can be directly given by

(A4) $${\bar{T}}_{y_{p}}=\frac{1}{n}\sum_{i=0}^{n-1}∥b_{i}-a_{i}∥.$$

Therefore, if we divide Equation (A3) by *n*, we get

(A5) $$\frac{1}{n}\sum_{i=0}^{n-1}∥b_{i}-a_{i}∥\leq \frac{p}{n}.$$

Finally if we replace the left term of Equation (A5) by the left term of Equation (A4), we get

(A6) $${\bar{T}}_{y_{p}}\leq \frac{p}{n}.$$

as expected. □
